# High Tree Species Diversity Promotes Thermal Enhancement Response of Microbial Carbon Use Efficiency

**DOI:** 10.1002/advs.76347

**Published:** 2026-07-03

**Authors:** Pengpeng Duan, Changqing Ye, Xinyi Yang, Chaoqun Wang, Wolfgang Wanek, Jian‐sheng Ye, Hongzhao Yuan, Hu Du, Kongcao Xiao, Xunyang He, Kelin Wang, Dejun Li

**Affiliations:** ^1^ Guangxi Key Laboratory of Karst Ecological Processes and Services Huanjiang Observation and Research Station For Karst Ecosystems Chinese Academy of Sciences Huanjiang China; ^2^ Institute of Subtropical Agriculture Chinese Academy of Sciences Changsha China; ^3^ School of Resources and Environmental Engineering Anhui University Hefei China; ^4^ College of Land Science and Technology Ministry of Agriculture and Rural Affairs Key Laboratory of Arable Land Conservation (North China) China Agricultural University Beijing China; ^5^ Division of Terrestrial Ecosystem Research Department of Microbiology and Ecosystem Science Center of Microbiology and Environmental Systems Science University of Vienna Vienna Austria; ^6^ State Key Laboratory of Grassland Agro‐Ecosystem College of Ecology Lanzhou University Lanzhou China

**Keywords:** microbial carbon use efficiency, soil organic carbon, species interactions, thermal adaptation, tree species diversity, warming

## Abstract

Predicting soil carbon dynamics under warming is constrained by limited understanding of microbial thermal adaptation, particularly whether microbial carbon use efficiency (CUE) can adapt to warming and how plant diversity modulates this response. Using soils from a natural tree species diversity gradient in a subtropical forest, we combined a 365‐day laboratory incubation with regular substrate amendment and ^18^O‐H_2_O labeling to quantify thermal responses of microbial respiration, growth, and CUE. High tree species diversity was associated with a strengthened compensatory thermal adaptation of microbial respiration and growth, effectively dampening their response to warming. Simultaneously, diversity promoted an enhanced thermal response of CUE, increasing microbial carbon retention capacity under warming. This dual regulation was mechanistically linked to a cascade of processes: higher tree species diversity was associated with lower soil organic matter stability (i.e., higher lability), minimizing bioenergetic costs of enzyme synthesis, facilitating a community‐wide shift toward r‐selected bacteria, and intensifying microbial competition as evidenced by network topology. Our findings reveal the potential of biodiversity to buffer soil carbon losses: conserving and restoring plant diversity can enhance soil capacity to mitigate climate change, both by reducing respiratory carbon losses and by increasing the potential for microbial carbon sequestration under warming.

## Abstract 2

How does forest biodiversity influence soil carbon loss under global warming? This study reveals that high tree species diversity dampens the warming response of microbial respiration while simultaneously enhancing carbon use efficiency. This dual regulation is linked to reduced soil organic matter stability and shifts toward opportunistic bacteria, highlighting biodiversity's role in modulating soil carbon retention.

## Graphical abstract

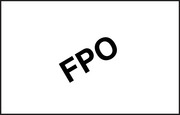

## 1 Introduction

Global climate warming, projected to increase surface temperatures by an additional 2.7°C–4.8°C by 2100 [1], poses a significant threat to the vast reservoir of carbon stored in soils. As the primary regulators of terrestrial carbon, soil microbes regulate the fate of this pool, and their response to warming will largely determine whether soils act as a net carbon source or sink [2]. The metabolic partitioning of assimilated carbon between respiratory CO_2_ release (catabolism) and biomass production (anabolism) is a pivotal control point [3]. This partitioning is quantified by microbial carbon use efficiency (CUE), which represents the fraction of carbon allocated to growth. A decline in CUE under warming would accelerate SOC loss and amplify positive carbon‐climate feedbacks, whereas a stable or enhanced CUE could mitigate these losses and help stabilize SOC stocks [4]. While Earth‐system models consistently predict accelerated microbial respiration with warming [5, 6], the magnitude of this feedback remains highly uncertain, demanding deeper mechanistic insights into the underlying microbial processes [7].

A major source of uncertainty lies in microbial thermal adaptation, the capacity of microbial communities to adjust their metabolic rates through physiological acclimation or compositional shifts [8, 9]. Field experiments frequently observe compensatory adaptation, where the initial stimulation of microbial respiration by warming attenuates over time, suggesting a potential for soils to mitigate carbon losses [10, 11, 12]. However, this focus on respiration overlooks the equally critical anabolic side of microbial metabolism. The thermal response of CUE is particularly contentious. While recent comparative analyses suggest that rates of thermal adaptation of microbial growth and respiration may converge across ecosystem types [13], the direction and magnitude of CUE responses remain highly variable, as studies report that warming can decrease [14, 15], increase [16, 17], or have no effect on CUE [18, 19]. This divergence in CUE responses may reflect the divergent temperature sensitivities of growth and respiration [20], as well as differences in substrate availability, experimental design, and the duration of warming, ultimately hindering a consensus on SOC‐climate feedbacks.

In natural ecosystems, microbial responses are embedded within a complex web of biotic interactions and resource fluctuations. A pivotal, yet largely overlooked, modulator of these responses is plant species diversity. Theoretical frameworks suggest that higher plant diversity could regulate microbial thermal responses through multiple synergistic pathways, including stoichiometric diversification [21], microclimate moderation [22], and facilitation of functional redundancy [23, 24]. Crucially, diversity may alter SOC stabilization, specifically the balance between mineral association and biochemical recalcitrance, which creates a fundamental trade‐off between physical protection and metabolic accessibility [25, 26]. However, empirical evidence linking these “diversity cascades” to the thermal adaptation of microbial metabolism, particularly CUE, is critically lacking.

Southwest China serves as an ideal model system to address this knowledge gap, functioning as both a global biodiversity hotspot [27] and a critical terrestrial C sink [28], yet highly susceptible to climate impacts [29]. Here, we conducted a year‐long soil incubation experiment using soils from a natural tree diversity gradient to test the overarching hypothesis that tree diversity governs the thermal adaptive strategies of soil microbes. We specifically hypothesized that: (1) higher tree species diversity strengthens the thermal compensation (down‐regulation, a stronger negative rate of increase with assay temperature) of microbial respiration; and (2) simultaneously promotes the thermal enhancement (up‐regulation, a stronger positive rate of increase with assay temperature) of microbial CUE. Furthermore, we posit that (3) these adaptive responses are mechanistically associated with diversity‐induced shifts in soil organic matter stability, which subsequently select for distinct microbial life‐history strategies (r‐ vs. K‐strategists) and alter the intensity of inter‐microbial competition. By integrating a natural diversity gradient with a controlled incubation using ^18^O‐labeling to precisely partition carbon fluxes, our study aims to unravel these complex interactions. The findings establish tree diversity as a critical modulator of microbial thermal adaptation via community restructuring, refining our understanding of how biodiversity underpins ecosystem resilience to climate warming.

## 2 Materials and Methods

### 2.1 Experimental Design and Field Sampling

The experimental site is situated within Mulun National Nature Reserve (107°54′01″–108°05′51″E, 25°07′01″–25°12′22″N) in Guangxi Province, southwest China. This region has a humid subtropical monsoon climate, with a mean annual temperature of 19.4°C and mean annual precipitation of 1500 mm. The lithology is limestone, dolomite, or their mixtures. The soil is calcareous, with the predominant soil types being Cambisols, Luvisols, and Leptosols (IUSS Working Group WRB, 2014). The selected forest was clear‐cut in the late 1950s and subsequently naturally regenerated. The spatial difference of tree species diversity was due to human disturbance during the early stage of forest development.

To capture this gradient, we established thirty 20 m × 20 m plots, ensuring a minimum buffer of 40 m between plots to maintain independence. These plots spanned a wide continuum of tree diversity, with Shannon's diversity index ranging from 0.65 to 3.46, across a modest elevation range (446–521 m). In each plot, a comprehensive inventory of dominant plant species was conducted (Data S1). Soil samples were collected from July 28 to August 1, 2020. Within each plot, we established 16 random sampling locations. At each location, we collected both the surface organic material (litter layer, 1–2 cm depth) and the underlying mineral soil to a depth of 10 cm using a 5 cm diameter corer. For each plot, the 16 mineral soil cores were pooled and homogenized to create a single composite sample. Visible roots and stones were meticulously removed using a 2‐mm mesh sieve. A subsample of fine roots (< 2 mm) was retained, cleaned, dried at 70°C for 48 h, and weighed to determine biomass and elemental content. Each composite soil sample was then partitioned into three subsamples for distinct analyses: one portion was stored at ‐20°C for DNA extraction, a second was kept fresh at 4°C for immediate microbial and biogeochemical assays and the incubation experiment, and the third was air‐dried for other physicochemical analyses.

### 2.2 Soil Physicochemical and Organic Matter Stability Analyses

We characterized a suite of soil properties following established protocols [30]. Soil pH was determined in a 1:2.5 soil‐to‐water suspension using a calibrated pH meter (FE20K, Mettler‐Toledo, Switzerland). Texture was assessed via laser diffraction (Malvern Mastersizer 2000, UK) after removal of organic matter and carbonates. Sand, silt and clay were defined by particle sizes of 2000–20, 20–2 and < 2 µm, respectively. SOC content was measured by the dichromate oxidation‐colorimetric method. Total nitrogen (N) content was analyzed using an elemental analyzer (EA 3000, EuroVector, Italy). Total phosphorus (P) content was measured colorimetrically using the ascorbic acid‐molybdate method after acid digestion in 0.5 m H_2_SO_4_.

To probe the mechanisms underlying microbial responses, we assessed soil organic matter (SOM) stability using both chemical and physical indices. The chemical recalcitrance of SOC was evaluated using ^13^C nuclear magnetic resonance (NMR) spectroscopy (see Methods S1). Briefly, chemical shifts were assigned to alkyl C, O‐alkyl C, aromatic C, and carbonyl C. The aliphaticity index (the proportion of aliphatic carbons, indicating less stable SOM) was calculated as the ratio of alkyl C to O‐alkyl C, whereas aromaticity (the proportion of aromatic carbons, indicating more stable SOM) was calculated as the ratio of aromatic C to the sum of alkyl C, O‐alkyl C, and aromatic C [31].

The degree of mineral protection was assessed through multiple metrics. We determined the abundance of different iron (Fe) and aluminium (Al) oxide forms, including pedogenic (Al_d_/Fe_d_, dithionite‐citrate‐bicarbonate extractable), poorly crystalline (Al_o_/Fe_o_, oxalate extractable), and organically complexed (Al_p_/Fe_p_, pyrophosphate extractable) forms, and exchangeable calcium/magnesium (Ca_exe_/Mg_exe_, 1 m ammonium acetate extractable)—with concentrations measured by ICP‐OES (Agilent, Santa Clara, USA). We also quantified the physical protection of SOC by fractionating soils into particulate organic matter (POC, >53 µm) and mineral‐associated organic carbon (MAOC, <53 µm), with details provided in Methods S2.

### 2.3 Experimental Incubation and Thermal Response Assays

To investigate microbial thermal adaptation, we conducted a year‐long incubation experiment. For 30 g composite soil samples from each plot, we prepared microcosms by adjusting the soil moisture to 60% of its water‐holding capacity (WHC). These soils were first pre‐incubated at 25°C in darkness for 90 days. This initial phase allowed microbial communities to recover from the disturbance of sampling and acclimate to laboratory conditions, a standard practice to eliminate the transient respiration flush associated with soil homogenization [24, 32, 33].

Following pre‐incubation, the soils from each plot were divided into three thermal treatments designed to simulate climate change scenarios. A reference treatment was maintained at the mean growing season temperature of 25°C (RT). A cooled treatment was incubated at 21°C (RT‐4°C), and a warmed treatment was incubated at 29°C (RT+4°C), reflecting a warming projection consistent with the IPCC SSP5‐8.5 scenario by 2100 [34, 35]. These temperatures (21°C, 25°C, and 29°C) were chosen to represent the mean growing season temperature of the study site (25°C) and realistic warming/cooling scenarios (±4°C) relevant to IPCC projections for the region. This resulted in 90 microcosms (30 plots × 3 thermal treatments) that were incubated for 365 days in darkness (Figure S1a,b). The duration enabled microbial metabolic acclimatization to temperature shifts (21°C and 29°C). The microcosms were sealed with Parafilm, which minimizes evaporation but allows gas exchange between the headspace of the vials and the ambient atmosphere. To prevent substrate depletion from confounding the temperature effects over the year‐long incubation, and to mimic the continuous supply of root exudates typical of the growing season in these evergreen forests, we amended the soils weekly with a small dose of glucose (0.8 mg C g^−1^ soil). Weekly glucose additions were calibrated to provide a baseline energy source, ensuring that microbial communities remained active without inducing massive priming effects, thus allowing us to isolate the thermal adaptation response [36, 37]. Moisture was rigorously maintained at 60% WHC by weekly weighing and adding sterile water. No significant difference in water loss rates was observed between thermal treatments due to the frequent adjustment.

At the end of the 365‐day adaptation period, we assessed the short‐term thermal response of microbial metabolism. From each microcosm, triplicate soil aliquots (0.4 g) were placed into 80 mL vials. To simultaneously measure microbial growth and respiration, we employed the ^18^O‐H_2_O labeling technique [38]. Each aliquot was treated with either ^18^O‐labeled water (97.0 at% ^18^O, to achieve 20% enrichment) or natural abundance ^16^O‐H_2_O as a control. These sub‐samples were then incubated for 24 h at three assay temperatures: 21°C, 25°C, and 29°C. This 24‐h assay duration was chosen to capture the active physiological response of the community while ensuring sufficient ^18^O incorporation into DNA for detection. This created a fully factorial design of 540 microcosms (30 plots × 3 adaptation treatments × 3 assay temperatures × 2 H_2_O labels). Headspace CO_2_ concentrations were measured by gas chromatography (GC‐7890A, Agilent, USA) to determine respiration rates. After the 24‐h assay, soils were immediately frozen at ‐80°C for DNA extraction by the DNeasy PowerSoil Pro kit (MoBio Laboratories, Carlsbad, CA, USA).

### 2.4 Calculation of Microbial Growth, Carbon Use Efficiency, and Adaptive Response

Microbial growth was determined by measuring the incorporation of ^18^O from labeled water into microbial DNA. The ^18^O abundance of purified DNA was analyzed using an elemental analyzer (FLASH2000HT) coupled to an isotope ratio mass spectrometer (MAT 253, Thermo Scientific, Bremen, Germany). Respiration, growth, and CUE were calculated following the equations in Zheng et al. [39]. To account for differences in microbial biomass, both growth and respiration rates were normalized to microbial biomass carbon (MBC) and are presented as mass‐specific rates (qGrowth and qRespiration) [40].

To quantify the degree of thermal adaptation, we calculated the magnitude of adaptive response (MAR) for each metabolic parameter (see Figure S1c for a schematic) [41]. For the warmed treatment, MAR was calculated as (A‐C)/(A‐B), and for the cooled treatment, as (A´‐C´)/(A´‐B), where A, A´, and B are the metabolic rates of the control soil (adapted to 25°C) when assayed at 29°C, 21°C, and 25°C, respectively, and C and C´ are the rates of the warmed and cooled soils assayed at their respective adaptation temperatures. For respiration and growth, MAR > 0 indicates a compensatory response (a dampening of the metabolic rate compared to the unadapted control), with higher values signifying stronger compensation. For CUE, MAR < 0 indicates an enhancing response (an increase in CUE compared to the unadapted control), with more negative values signifying a stronger enhancement.

Although the MAR index is widely employed to quantify thermal adaptation and facilitate cross‐site comparisons [41, 42], its inherent methodological assumptions warrant careful consideration. Specifically, MAR assumes linear metabolic shifts across the assayed temperature intervals, whereas actual microbial responses frequently exhibit nonlinearity near thermal optima. Additionally, utilizing the 25°C‐adapted community as an “unadapted” baseline presupposes a stable physiological reference, and calculating MAR as a ratio of differences can propagate mathematical uncertainties when the denominator is small. Consequently, MAR values should be strictly interpreted as robust indicators of relative directional shifts in microbial metabolism, rather than absolute physiological constants.

### 2.5 Microbial Biomass, Enzyme Activities and Community Analysis

Microbial biomass carbon (MBC) was measured using the chloroform‐fumigation extraction method on soils collected at the end of the preincubation and 365‐day incubation. Briefly, 10 g of fresh soil underwent chloroform fumigation (24 h) followed by 0.5 M K_2_SO_4_ extraction for the determination of dissolved organic C (DOC). DOC from paired non‐fumigated samples was also quantified via Total Organic Carbon Analyzer (TOC‐VWP, Shimadzu, Japan) to serve as a direct index of end‐of‐incubation substrate availability. MBC was calculated as ΔDOC_fumigated‐nonfumigated_ × 0.45.

Soil extracellular enzyme activities (EEA), including α‐Glucosidase (AG), β‐D‐glucosidase (BG), β‐D‐cellobiosidase (CBH), L‐leucine aminopeptidase (LAP), β‐*N*‐acetylglucosaminidase (NAG) and acid phosphatase (AP), were measured fluorometrically [43]. In brief, soil slurries (1.0 g in 100 mL 50 mm Tris, pH 7.4) were homogenized (2 min vortex), then dispensed into microplates (200 µL slurry + 50 µL 200 µm substrate per well). Substrate use was 4‐methyl‐umbelliferyl (MUB)‐conjugates (AG: MUB‐α‐glucoside; BG: MUB‐β‐glucoside; CBH: MUB‐cellobioside; NAG: MUB‐N‐acetylglucosaminide) and 4‐methylcoumaryl‐7‐amide (MCA)‐L‐leucine (LAP). After 4 h incubation (20°C, dark), reactions were quenched with 0.5 m NaOH (10 µL per well) prior to fluorescence quantification (365 nm excitation and 450 nm emission wavelength). Enzyme activities are expressed as nmol h^−1^ g^−1^. The sum of AG, BG, and CBH activities denotes the total activity of C–acquiring hydrolases (EEA_C_), and the sum of LAP and NAG activities represents the total activity of N–acquiring hydrolases (EEA_N_). Enzyme activities are expressed as nmol h^−1^ g^−1^.

Total microbial DNA was extracted from soils at the end of the incubation using the DNeasy PowerSoil Pro kit (MoBio Laboratories, Carlsbad, CA, USA). The V3–V4 region of the bacterial 16S rRNA gene and the fungal ITS1 region were amplified using primers 338F/806R and ITS1/ITS2, respectively. Amplicons were sequenced on an Illumina MiSeq platform (Illumina Inc., California, USA). Raw sequences were processed using the QIIME2 pipeline to cluster Operational Taxonomic Units (OTUs) at a 97% similarity threshold. See Methods S3 for DNA extraction and sequencing analysis.

### 2.6 Co‐Occurrence Network Construction and Life‐History Strategy Characterization

To elucidate the intricate inter‐taxa relationships within microbial communities and identify ecologically relevant clusters, co‐occurrence networks were constructed. Prior to network generation, data were meticulously filtered to minimize spurious correlations, retaining only OTUs present in at least 80% of the soil samples. Spearman correlation coefficients were computed for all pairwise associations among the filtered OTUs. Only correlations with an absolute value of r>0.65 and a false discovery rate (FDR) corrected p‐value <0.05 were used for network construction, ensuring robust and significant interactions. Ecological clusters, defined as groups of taxa exhibiting strong mutual interactions, were identified using a greedy modularity optimization algorithm, which quantifies the degree to which the network is partitioned into distinct modules. For each identified module, its relative abundance was calculated by averaging the z‐transformed relative abundances of all constituent taxa. We calculated a suite of network topological indices (e.g., nodes, edges, connectance, degree, clustering coefficient, vulnerability, clusters, diameter, average path length, betweenness centrality, and centralization) and identified ecological clusters (modules) using a greedy modularity optimization algorithm via the “*ggClusterNet*” and “*igraph*” packages.

Cohesion, a critical metric in microbial ecology, was used to quantify the structural properties of microbial association networks, reflecting the strength of species interactions and their impact on community stability. It is typically differentiated into positive cohesion (representing facilitative interactions like mutualism) and negative cohesion (representing inhibitory interactions like competition or antagonism). Network stability was quantitatively assessed using the ratio of |Negative|: Positive cohesion [44], where higher values signify greater stability and competitive dominance.

We classified microbial communities based on life‐history strategies (r‐ vs. K‐strategists) at the phylum level following Duan et al. [25]. The ratio of K to r species was calculated after summing the oligotrophic and copiotrophic bacterial or fungal phyla, respectively. Additionally, we estimated the community‐weighted mean rRNA operon (*rrn*) copy number, a trait‐based indicator of bacterial life‐history strategy. Higher *rrn* copy numbers are associated with faster growth potential and r‐selected strategies [45].

### 2.7 Statistical Analyses

Microbial α‐diversity metrics, specifically Shannon and Chao1 indices, were calculated using QIIME2. To assess differences in microbial community structure (β diversity) among the cooled, control, and warmed conditions, nonmetric multidimensional scaling (NMDS) ordination was performed based on Bray‐Curtis distance matrices. Three non‐parametric multivariate analyses of dissimilarity, including multiple‐response permutation procedure (MRPP), analysis of similarity (ANOSIM), and Adonis, were conducted using the Bray‐Curtis distance and R “*vegan*” package to statistically test for significant community shifts.

Response ratios (RR) were calculated by taking the natural logarithm of the ratio of measured variables in the warmed or cooled treatments relative to those in the control. These variables included extracellular enzyme activities, microbial α‐diversity, network complexity, life‐history strategies, and both negative and positive cohesions under thermal treatments. Subsequently, regression analysis was performed to investigate the relationships among RR, MAR, and tree species diversity.

To identify the influence of soil physicochemical properties and organic matter stability on the magnitude of adaptation response of microbial metabolic parameters under warmed or cooled conditions, random forest modeling was applied using the “*randomForest*” package. Variable importance was assessed by the increase in mean squared error (%Increase MSE) resulting from random permutation of each variable, with a larger MSE increase indicating greater predictor importance.

Based on the key drivers identified from the random forest model, structural equation modeling (SEM) was performed to evaluate the hypothesized structural pathways linking soil physicochemical properties, organic matter stability, RR of extracellular enzyme activities, K:r ratio, *rrn*, and |Negative|: Positive cohesion on magnitude of adaptation response of microbial metabolic parameters. Model fit was progressively refined by adding or removing relationships among variables based on modification indices from the prior model (Figure S2). The SEM was established in AMOS 21.0 (Amos Development Corporation, Chicago, IL, USA), while all other statistical analyses were conducted in R version 4.2.0 (R Core Team, 2022).

## 3 Results

### 3.1 Tree Species Diversity Drives Divergent Thermal Adaptation of Microbial Metabolism

**FIGURE 1:**
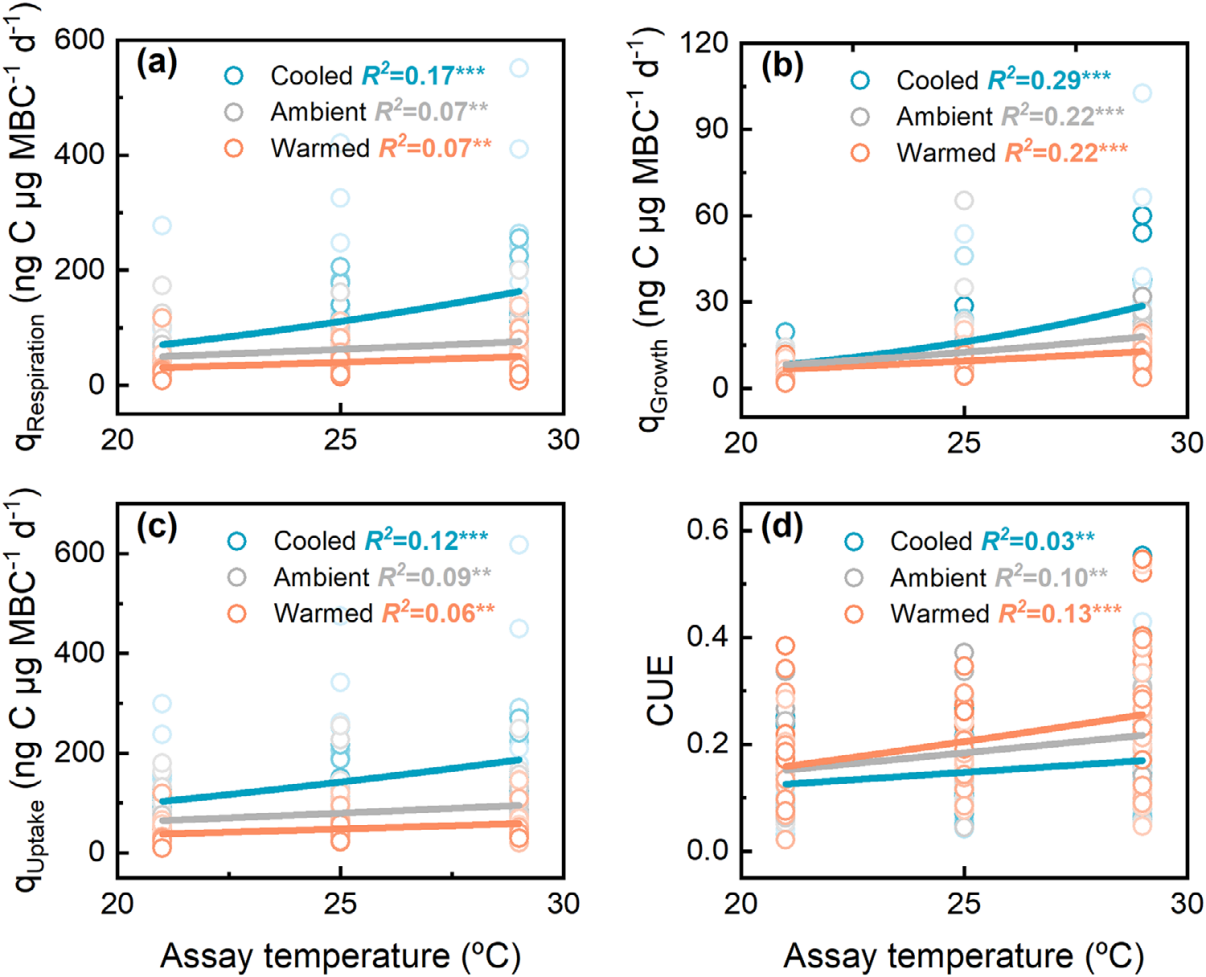
Thermal response of microbial metabolic parameters to assay temperature (21°C, 25°C and 29°C). Growth, respiration, and carbon uptake are expressed per unit of microbial biomass. The relationships between microbial metabolic parameters and assay temperature were fitted to exponential models. The R^2^ values and significance levels indicate the goodness‐of‐fit for each individual exponential model. Significance is indicated by ^*^ *p* < 0.05; ^**^ *p* < 0.01; and ^***^ *p* < 0.001.

Across all treatments, mass‐specific rates of respiration, growth, and C uptake increased with the assay temperature (Figure 1a–d). However, the magnitude of this response was contingent on the long‐term adaptation temperature. Soils adapted to cooler conditions (21°C) exhibited the strongest temperature sensitivity, while soils adapted to warmer conditions (29°C) showed dampened responses for respiration, growth, and uptake. This pattern provides clear evidence for a compensatory thermal adaptation in microbial catabolic and anabolic pathways. In contrast, microbial CUE displayed an enhancing thermal adaptation; its positive response to assay temperature was most pronounced in the soils adapted to warming (Figure 1d).

**FIGURE 2:**
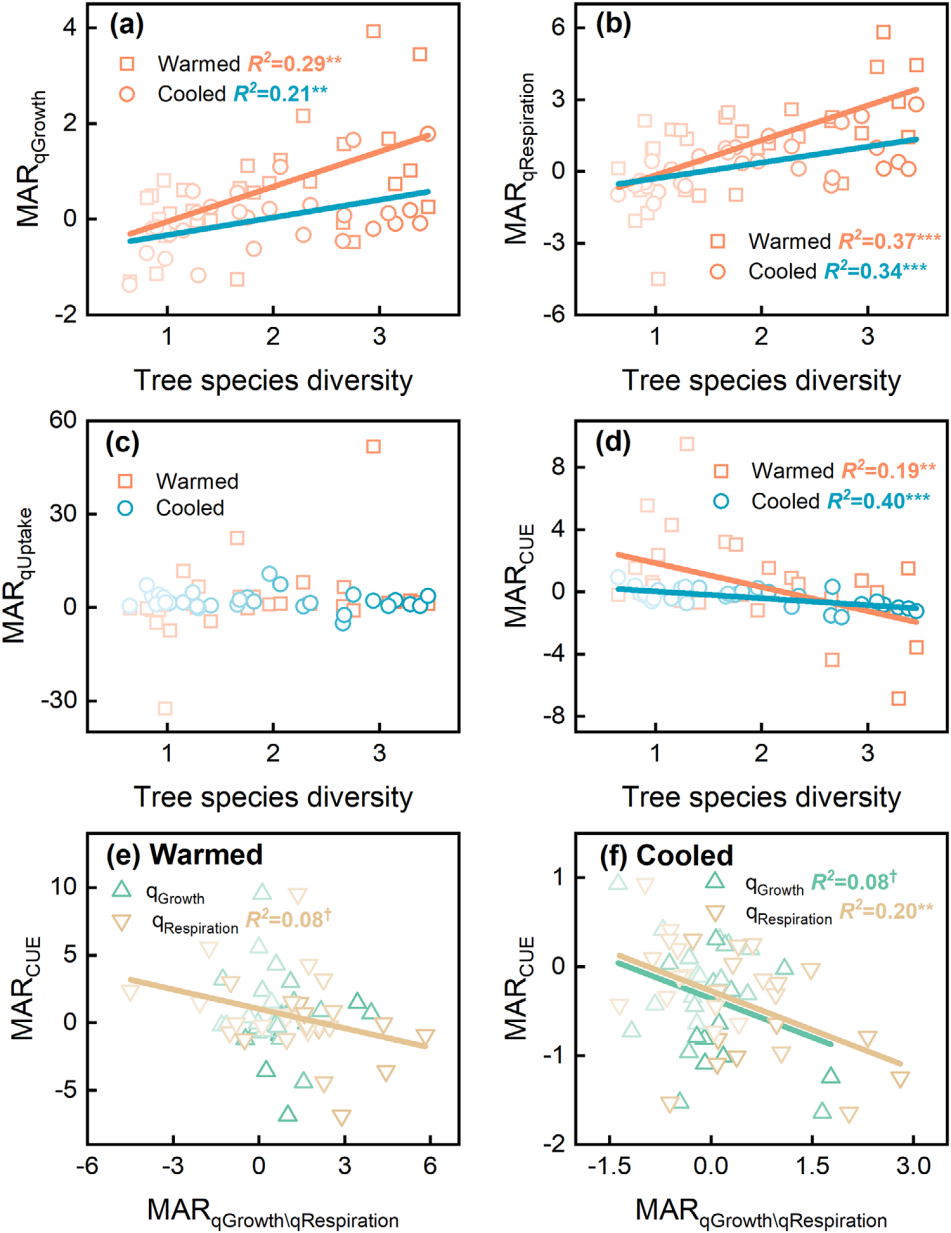
The effects of tree species diversity on the magnitude of adaptive responses (MAR) of microbial metabolic parameters (a–d), and relationships between the magnitude of adaptive responses (MAR) of microbial CUE, mass‐specific growth and respiration (e‐f). Significance is indicated by ^*^ *p* < 0.05; ^**^ *p* < 0.01; and ^***^ *p* < 0.001. For qrespiration and qgrowth, MAR > 0 indicates compensatory adaptation (dampened response relative to the unadapted control); for CUE, more negative MAR indicates greater enhancement.

Crucially, the strength of these adaptive responses was systematically linked to the diversity of the overlying tree community. Increasing tree species diversity was significantly associated with a stronger compensatory adaptation of microbial respiration and growth. This was evident as a positive correlation between diversity and the MAR for both q_Growth_ (*R^2^* = 0.29, *p*<0.01 in warmed soils) and q_Respiration_ (*R^2^* = 0.37, *p*<0.001 in warmed soils) (Figure 2a,b). Since MAR > 0 indicates a dampening of the metabolic rate relative to the unadapted control, this demonstrates that microbes in more tree‐diverse forests were better able to downregulate their metabolic rates.

The most striking finding was the opposing effect of diversity on CUE. Higher tree species diversity was strongly and negatively correlated with the MAR of CUE (*R^2^* = 0.40, *p*<0.001 in cooled soils; *R^2^* = 0.19, *p*<0.01 in warmed soils) (Figure 2d). Since a more negative MAR value signifies a greater deviation above the unadapted baseline, this unequivocally demonstrates that higher diversity was linked to a more intense positive, enhancing thermal adaptation of microbial CUE. The thermal adaptation of CUE was more closely coupled with the adaptation of respiration than growth, particularly under cooling, suggesting that the regulation of catabolic pathways is a key determinant of overall efficiency (Figure 2e,f).

### 3.2 Regulation of Tree Species Diversity on Thermal Response of Soil Microbial Metabolism

**FIGURE 3:**
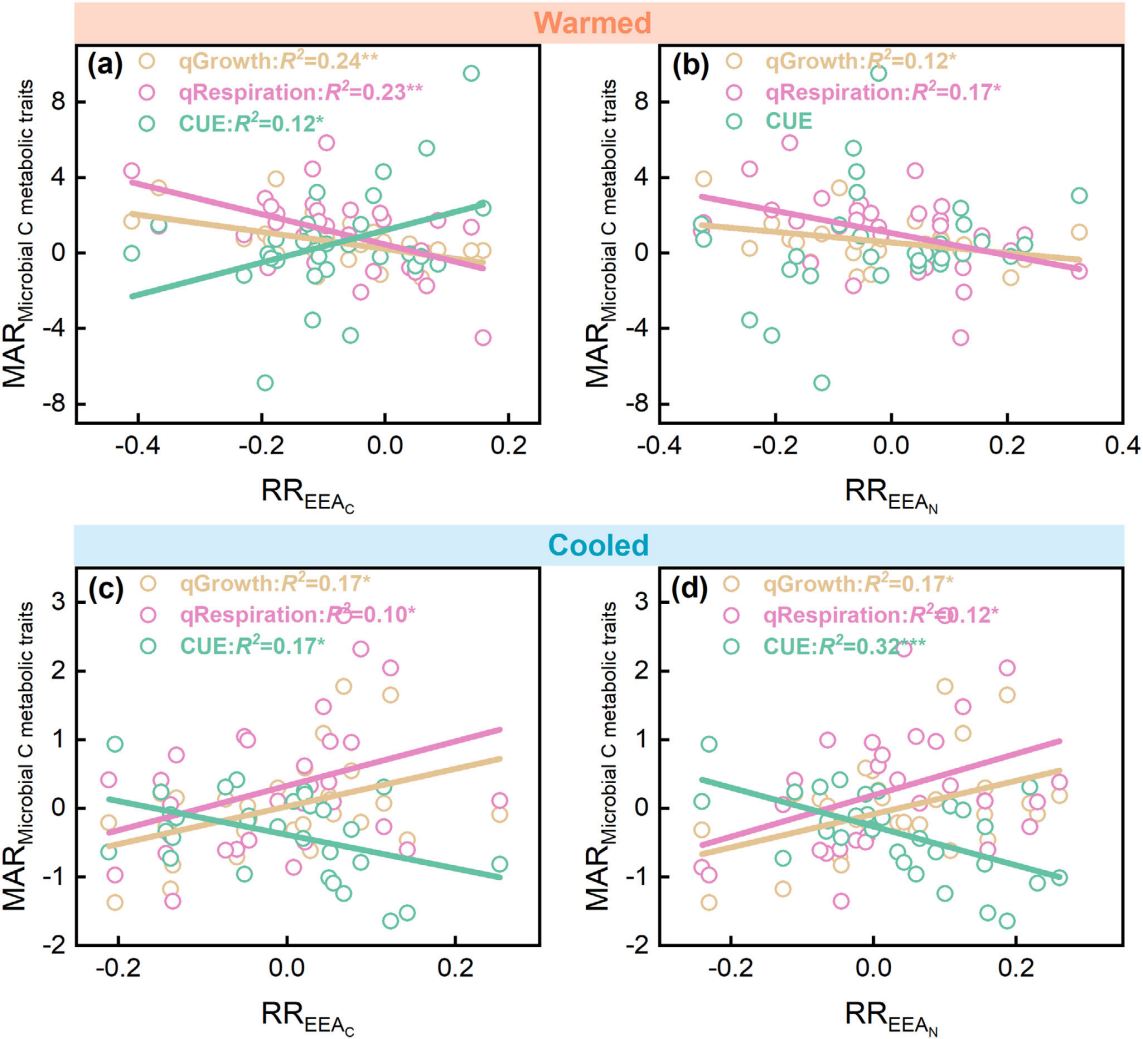
Relationships between the magnitude of adaptive responses (MAR) of microbial metabolic parameters and the response ratios (RR) of C‐acquiring (EEA_C_) and N‐acquiring (EEA_N_) enzyme activities under (a, b) warmed and (c, d) cooled conditions. Each point represents the response ratio of the summed total activity of the respective enzyme group: EEA_C_ represents the sum of α‐glucosidase, β‐glucosidase, and β‐D‐cellobiosidase activities, and EEA_N_ represents the sum of L‐leucine aminopeptidase and β‐N‐acetylglucosaminidase activities. Significance is indicated by ^*^ *p* < 0.05; ^**^ *p* < 0.01; and ^***^ *p* < 0.001.

Response ratios of potential extracellular enzyme activities, reflecting the size of the active enzyme pool accumulated over the 365‐day incubation period, showed that under warmed conditions, MAR_qGrowth_ and MAR_qRespiration_ were negatively associated (*p* < 0.05) with the response ratios of C‐acquiring (RR_EEAC_) and N‐acquiring (RR_EEAN_) enzyme activities (Figure 3a,b). This implies that a stronger compensatory response in respiration and growth coincided with a reduced temperature sensitivity of enzyme activities. Conversely, MAR_CUE_ showed a positive association with RR_EEAC_ (Figure 3a), suggesting that an enhanced CUE adaptation was linked to greater temperature sensitivity of C‐acquiring enzymes. Under cooling conditions, these relationships dramatically reversed: MAR_qGrowth_ and MAR_qRespiration_ exhibited positive correlations (*p* < 0.05) with RR_EEAC_ and RR_EEAN_, while MAR_CUE_ was negatively associated with both RR_EEAC_ and RR_EEAN_ (Figure 3c,d). This reversal in the direction of enzyme activity response ratios between warmed and cooled treatments suggests a context‐dependent shift in the balance between enzyme investment and substrate access under different thermal regimes.

**FIGURE 4:**
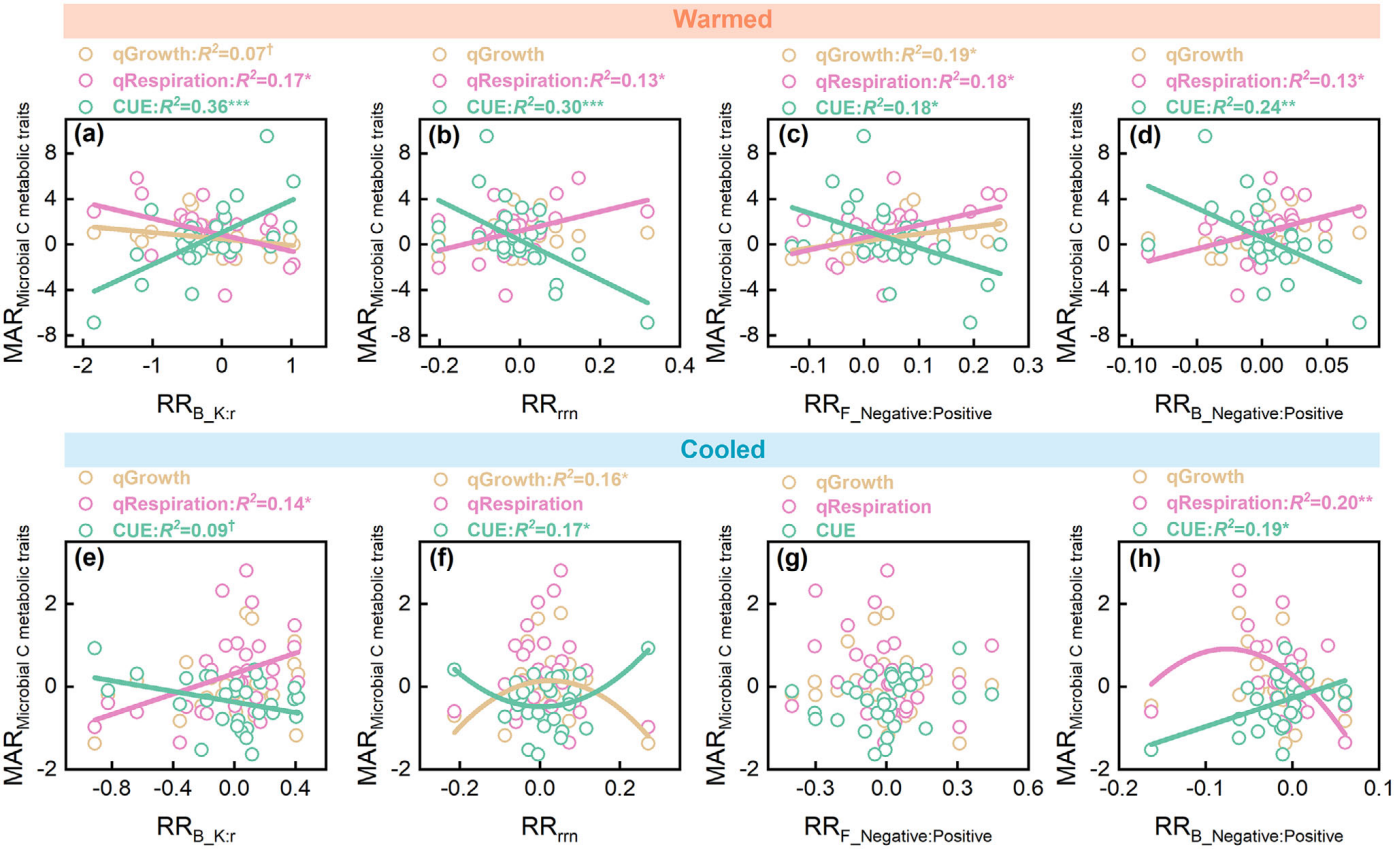
Relationships between the magnitude of adaptive responses (MAR) of microbial metabolic parameters and the response ratios (RR) of bacterial K:r ratios, *rrn* copy numbers, and microbial network stability (represented by |negative|: positive cohesion ratios). Significance is indicated by ^*^ *p* < 0.05; ^**^ *p* < 0.01; and ^***^ *p* < 0.001.

In the warmed treatment (Figure 4a–d), MAR_qGrowth_ displayed a weaker, negative relationship with the bacterial K:r ratio response ratio (RR_B_K:r_) but increased with the fungal negative: positive cohesion ratio response ratio (RR_F_Negative: Positive_). MAR_qRespiration_ decreased with RR_B_K:r_ but was positively (*p* < 0.05) related to both the *rrn* copy number response ratio (RR_rrn_) and the microbial negative: positive cohesion ratio response ratio (RR_Negative: Positive_). In contrast, MAR_CUE_ exhibited a positive (*p* < 0.05) association with RR_B_K:r_ and significant negative (*p* < 0.05) correlations with both RR_rrn_ and microbial RR_Negative: Positive_. These patterns strongly suggest that the thermal enhancement of CUE in warmed soils is intricately linked to shifts toward r‐strategist bacterial communities and intensified competitive interactions within the microbial network. Under cooling conditions (Figure 4e–h), MAR_qRespiration_ showed a modest positive association with RR_B_K:r_, while MAR_CUE_ experienced a marginal decline. Notably, cooled soils revealed a significant unimodal (quadratic) relationship for both MAR_qGrowth_ and MAR_CUE_ with RR_rrn_. Additionally, the bacterial negative: positive cohesion ratio response ratio (RR_B_Negative: Positive_) predicted an increase in MAR_CUE_ and a curved response in MAR_qRespiration_, suggesting that colder conditions may favor microbial carbon allocation strategies when antagonistic interactions become more prevalent.

**FIGURE 5:**
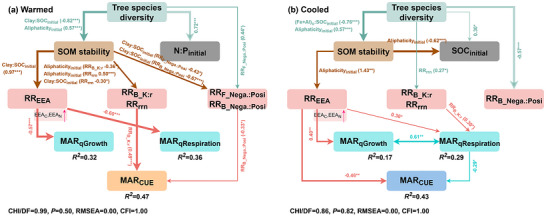
Structural equation models (SEM) assessing the effects of tree species diversity and other multiple ecological predictors on the magnitude of adaptive responses (MAR) of microbial metabolism for (a) the warmed treatment and (b) the cooled treatment. Principal component analysis (PCA) was used to create multivariate indices for C and N‐acquiring enzyme activities. The first principal component (PC1) of site scores was used in the SEM analysis, with “↑” and “↓” indicating a positive or negative correlation between the factors and the first component from the PCA, respectively. Each box represents a group of variables, and each arrow summarizes all the significant effects between all the variables of two boxes. Pathway relations are drawn with simple arrows, and arrow widths are scaled to the sum of the absolute standardized effect sizes of significant relations between all variables of the two nodes. Only significant relationships between variables are indicated, and significance levels are shown at ^*^ *p* < 0.05, ^**^ *p* < 0.01, ^***^ *p* < 0.001. *R^2^* denotes the proportion of variance explained. SOM, soil organic matter; EEA, extracellular enzymes activity; nega.:posi., |negative: positive cohesion ratio|.

We integrated these findings into SEM to evaluate the hypothesized structural pathways linking tree diversity to microbial adaptation (Figure 5). The model indicated that the influence of tree species diversity was primarily mediated through its relationship with SOM stability. Under warming conditions (Figure 5a), higher tree species diversity was associated with lower SOM stability (i.e., reflecting a negative path coefficient toward clay: SOC and a positive one toward aliphaticity). This lower SOM stability was associated with a down‐regulation of RR_EEA_, correlating with a shift toward copiotrophic life strategies (reduced RR_B_K:r_ and stimulated RR_rrn_) and more intense competition (higher RR_Nega: Posi_). Ultimately, this statistical cascade explained the variance in the divergent metabolic adaptations: the dampened enzyme response covaried with the compensatory adaptation of microbial growth and respiration, whereas the combination of intensified competition and the rise of r‐strategists was linked to the enhancement of CUE.

Under cooling conditions (Figure 5b), higher tree species diversity, acting via reduced SOM stability, was associated with greater enzyme sensitivity to cooling. This pathway positively correlated with MAR_qGrowth_ and MAR_qRespiration_, while negatively correlating with MAR_CUE_. Additionally, increased tree species diversity was directly linked to a shift in microbial life strategies, which in turn corresponded to variations in MAR_qRespiration_. The enhancement of CUE under cooling was thus modeled primarily as an indirect correlate of this respiratory adaptation.

## 4 Discussion

### 4.1 Tree Species Diversity Enhances Respiratory Compensation and Carbon Use Efficiency

Our study reveals a critical, yet previously unrecognized, dimension of how terrestrial ecosystems may respond to climate warming. We demonstrate that tree species diversity governs the thermal adaptation of the soil microbial engine, orchestrating a dual response that buffers against soil carbon loss. In more diverse forests, microbes simultaneously dampen their respiratory response to warming while paradoxically enhancing their CUE. This finding challenges the conventional focus on respiratory compensation alone and provides a novel mechanistic framework for understanding how biodiversity underpins ecosystem resilience. By integrating field‐based diversity gradients with controlled isotopic tracing, we unraveled a mechanistic link: from tree diversity to soil organic matter stability, and onward to microbial life‐history strategies and interactions, which ultimately dictate the metabolic fate of soil carbon in a warmer world.

A pivotal aspect of our experimental design was the weekly addition of glucose (0.8 mg C g^−1^ soil). While this amendment could theoretically drive a uniform selection for r‐strategists across all treatments, the significant and divergent responses observed along the tree diversity gradient argue against a simple experimental artifact. Instead, the glucose addition likely standardized substrate availability, unmasking the inherent adaptive potential of the microbial communities shaped by the long‐term legacy of tree diversity. By alleviating severe carbon starvation, our design allowed the distinct metabolic capabilities of diversity‐selected communities to manifest. Thus, our findings reflect the “potential” CUE response in active rhizosphere hotspots or periods of high root exudation, where microbes are not strictly C‐limited. This reveals the potential metabolic trajectory of soil microbiomes under future climate scenarios where root exudation rates may increase due to CO_2_ fertilization and extended growing seasons. The fact that high‐diversity soils exhibited stronger adaptation even under this uniform C supply reinforces the conclusion that the legacy of plant diversity fundamentally alters microbial physiological potential.

### 4.2 Reduced Soil Organic Matter Stability Fosters Microbial Bioenergetic Efficiency

A central and counter‐intuitive finding of our work is that the thermal enhancement of microbial CUE in highly diverse forests is linked to lower soil organic matter stability. Specifically, we found that high tree diversity was associated with lower mineral protection (Clay: SOC) and greater chemical lability (aliphaticity) (Figure 5). In subtropical karst ecosystems, soils are typically shallow and reactive clay minerals are inherently limited. Consequently, massive organic inputs from highly diverse tree communities rapidly saturate these available mineral surfaces [46]. Surplus carbon thus accumulates primarily as unprotected, labile particulate organic matter, thereby driving down the overall relative mineral stabilization (e.g., lower Clay: SOC). Concurrently, the greater chemical lability (increased aliphaticity) in tree species‐rich plots likely stems from a combination of enhanced root exudation and elevated soil pH. Significantly higher fine root biomass in diverse stands provides continuous inputs of aliphatic‐rich root litter and exudates [47, 48], constantly replenishing the labile SOM pool. Moreover, tree diversity‐driven increases in soil pH can amplify the negative surface charge on reactive minerals, thereby inhibiting the adsorption of aromatic compounds [49] and promoting their desorption or dissolution via enhanced solubility [50]. This selective retention of aliphatics shifts the overall SOM pool toward higher chemical lability (Figures S3 and S4).

Prevailing biogeochemical paradigms suggest that reduced SOM protection accelerates carbon loss under warming [51]. However, our SEM analysis (Figure 5) clarifies that in high tree diversity soils, this less stable organic matter structure acts as an adaptive energetic advantage. High tree species diversity dismantles C and N constraints by providing abundant labile substrates, while reduced chemical recalcitrance and lower mineral protection increase native SOC accessibility. Together, these factors lower the activation energy required for microbial metabolism and reduce the energetic incentive to prime older, recalcitrant carbon [52]. Therefore, while high tree species diversity renders SOC physicochemically more accessible, it simultaneously stabilizes this pool biologically by shifting microbial metabolism away from native SOC decomposition.

From a bioenergetic perspective, accessing labile, unprotected carbon requires significantly less extracellular enzymatic investment than mining mineral‐protected or recalcitrant pools. Consequently, microbes in diverse plant soils are able to down‐regulate the production of energy‐intensive extracellular enzymes (Figure 3; Figure S5). While experimental glucose amendments globally suppress enzyme production via substrate feedback, the pronounced, diversity‐dependent down‐regulation we observed highlights a synergistic cost‐saving strategy driven by the highly accessible native SOM. By minimizing resource allocation to enzyme synthesis and maintenance respiration, microbes channel a greater proportion of assimilated carbon‐ from both native SOM and added glucose toward biosynthesis [53]. This recasts our understanding of SOM stability: although lower mineral protection inherently reduces the passive persistence of SOC, it simultaneously cultivates a metabolically efficient, r‐selected microbial community that maximizes biomass (and potential necromass) production. Ultimately, this represents a fundamental paradigm shift where the primary mechanism of carbon sequestration transitions from the physical protection of initial inputs to the maximization of microbial conversion efficiency.

Our results strongly suggest that the observed “adaptation” over 365 days is primarily linked to species sorting (community reassembly) rather than physiological acclimation of individual taxa (Figures S6 and S7). The transition toward a community dominated by faster‐growing, r‐selected bacteria in warmed, plant‐diverse soils was unequivocal, supported by both taxonomic classifications and trait‐based *rrn* copy number analysis (Figure 4; Figure S8). These copiotrophic taxa are inherently adapted to capitalize on resource‐rich environments [54]. While classically viewed as energetically “wasteful” under oligotrophic conditions, copiotrophs can rapidly convert available carbon into biomass with remarkably high efficiency when resource constraints are alleviated [55]. In our study, the combination of glucose amendment and readily degradable soil organic matter in diverse soils provided the optimal context for these opportunistic microbes to thrive, maximizing their growth yield and driving a community‐wide shift toward higher CUE. Warming acts as a powerful selective filter (Figure S9), and the degradable soil organic matter in diverse soils provides the ideal arena for these opportunistic microbes to thrive, effectively accelerating a community‐wide shift toward higher CUE.

Furthermore, we provide novel evidence that the intensification of microbial interactions is a key component of thermal adaptation. To quantify the intensity of microbial competitive interactions, we used the ratio of absolute negative to positive network cohesion (|Negative|: Positive cohesion). This metric increases when competitive interactions dominate the co‐occurrence network. Increased negative cohesion within microbial networks under warming (reflected by a higher RR_Negative: Positive_) was associated with stronger compensatory adaptation of respiration and growth, as well as enhanced CUE adaptation (Figure 4; Figure S10). This likely reflects intense competition for limiting nutrients (N and P) in a C‐rich environment (fueled by both high‐quality litter legacies and glucose addition). The competitive dominance observed in diverse forests is conceptually analogous to the “hunger games” hypothesis [45], originally proposed for marine bacterial communities, which posits that resource‐rich environments amplify competitive exclusion and selectively favor the most metabolically efficient taxa. While direct validation in terrestrial warming contexts is still limited, recent evidence from soil antagonism studies [56] and bacterial–fungal competition research [57] supports the principle that resource enrichment intensifies competitive interactions and promotes efficient resource use in soil microbial communities. However, we acknowledge that increased negative cohesion in co‐occurrence networks may also reflect divergent niche preferences (environmental filtering) rather than direct antagonistic interactions, though the outcome, dominance of efficient taxa, remains consistent. These network‐driven effects suggest that microbial community interactions function as a crucial regulatory switch, dampening carbon losses through competitive exclusion and efficiency selection in diverse forests.

To rule out substrate depletion as an artifact of the observed compensatory responses, we quantified DOC post‐incubation. DOC significantly increased with tree diversity across all temperatures, peaking under warmed conditions (29°C; Figure S11). This confirms that microbes in warmed, diverse soils were not resource‐limited. Instead, abundant labile substrates facilitated genuine biological down‐regulation of mass‐specific respiration and enhanced CUE. However, whether this adaptive advantage persists over multi‐decadal warming remains uncertain. The dominance of copiotrophs and reduced enzyme investment rely on continuously accessible substrates. Although diverse forests provide sustained litter inputs, prolonged warming could eventually deplete labile carbon, constraining the competitive edge of r‐strategists [17, 58]. This underscores the critical need for multi‐year field experiments tracking microbial trajectories alongside SOC stocks.

### 4.3 Ecological Implications for Soil Carbon Cycling and Study Limitations

**FIGURE 6:**
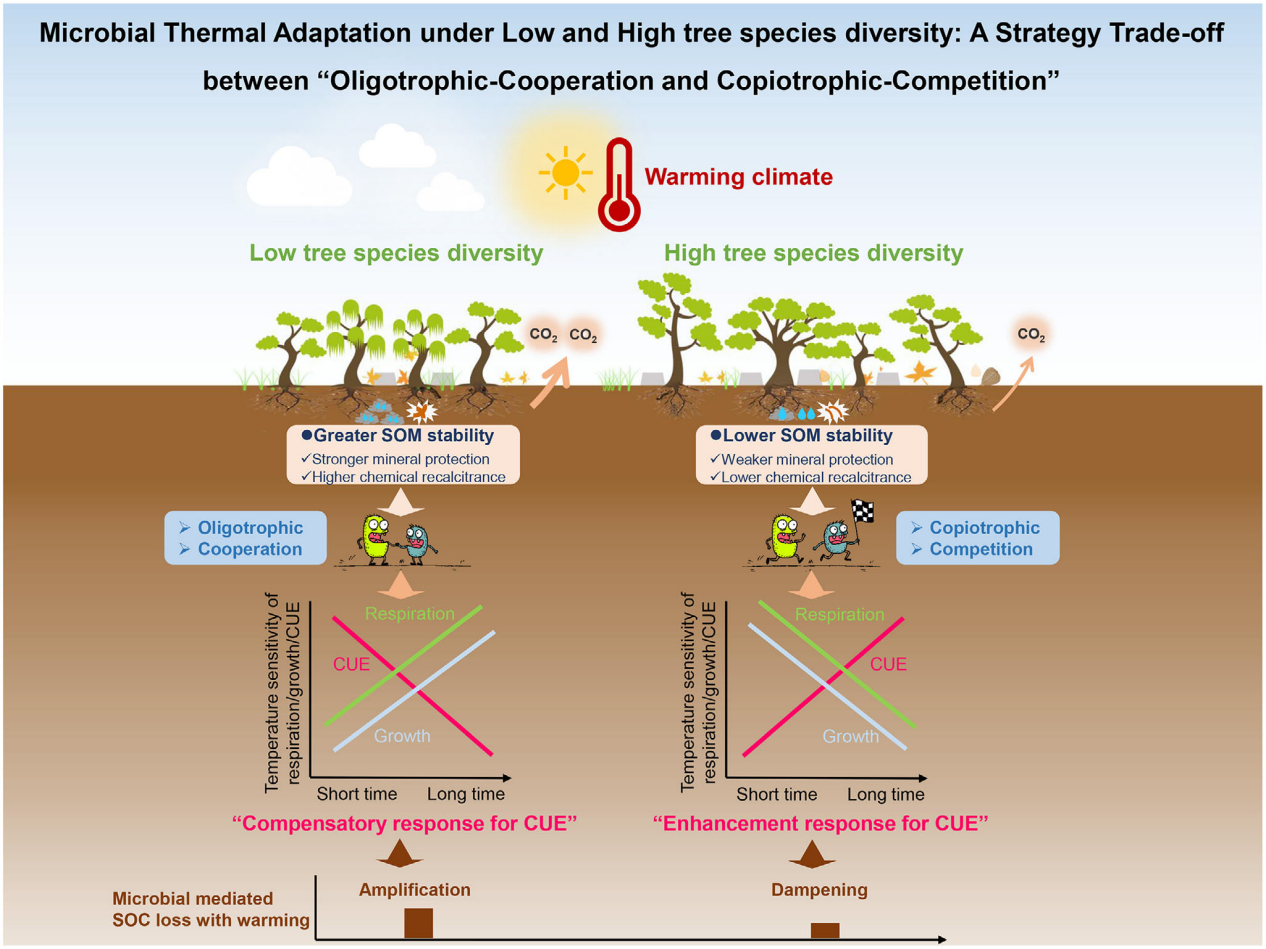
Conceptual diagram illustrating the control of tree species diversity on the thermal adaptation of soil microbial metabolic parameters, and the predicted impacts on soil carbon loss. In species‐rich forests, reduced soil organic matter (SOM) stability, due to weaker mineral protection and lower chemical recalcitrance, enhances microbial responses to warming. The response ratio of carbon and nitrogen‐acquisition enzyme activity decreases, as less extracellular investment is needed. Bacterial communities shift toward copiotrophic selection, evidenced by a lower response ratio of the K:r ratio and an elevated response ratio of *rrn* copy number, enabling rapid substrate exploitation under warming. The response ratio of the negative: positive cohesion ratio rises, reflecting intensified competitive interactions with temperature. Collectively, these adjustments, including diminished enzyme allocation, accelerated life‐history strategies, and stronger competition, synergistically boost the thermal adaptation of microbial carbon use efficiency (CUE), therefore dampening warming‐induced SOC losses and enhancing ecosystem carbon sequestration potential.

The implications of our findings for soil carbon cycling under global warming are profound. Current models, which often project substantial carbon losses from soils, may be underestimating the buffering capacity of biodiverse ecosystems. Our results suggest a dual mechanism by which tree species diversity can counteract warming‐induced carbon emissions (Figure 6). First, it promotes the compensatory adaptation of respiration, reducing direct CO_2_ efflux. Second, and perhaps more importantly, it enhances the thermal adaptation of CUE, channeling more carbon into microbial biomass. Since microbial necromass is a primary precursor for stable SOC formation, this enhanced anabolic efficiency implies a greater potential for long‐term carbon sequestration, provided that this new biomass can be subsequently stabilized. Future studies quantifying microbial necromass accumulation are needed to validate this potential.

Although our controlled incubations demonstrate a clear tree diversity‐driven enhancement of CUE thermal adaptation, field conditions with fluctuating moisture, root inputs, and aggregate turnover may alter the realized magnitude of this effect. The one‐year incubation period, while sufficient to capture microbial reassembly, still represents a simplified microcosm. As noted, the weekly glucose amendment, while essential to prevent substrate depletion, may have partially promoted copiotrophic communities across all plots, potentially amplifying the diversity‐associated shifts toward r‐strategists. Although identical glucose doses were applied across all diversity levels (making glucose dose itself uninformative about diversity effects), the possibility that glucose × diversity interactions contributed to the observed community shifts cannot be entirely excluded. Future research should prioritize multi‐year field warming experiments without external substrate addition to validate if these potential efficiency gains translate into realized soil carbon stocks under varying natural substrate regimes. Furthermore, integrating more advanced isotopic tracers beyond ^18^O‐H_2_O, such as ^2^H‐vapor‐FAME (fatty acid methyl ester)‐SIP, could provide finer‐scale insights into how warming influences the precise allocation of carbon to biomass growth versus the synthesis of storage compounds [59].

It is also important to note that the mechanisms identified here are anchored in the specific geochemistry of subtropical karst soils, where Ca‐bridging rather than Fe/Al‐oxide organo‐mineral associations are the primary means of SOM protection [46, 60]. In acidic tropical or boreal forests dominated by Fe/Al‐bound organic matter [61, 62], higher plant diversity may enhance rather than reduce SOM mineral protection, potentially dampening or reversing the CUE‐enhancement pathway described here. Similarly, in permafrost‐affected soils and forests with fundamentally different tree family compositions, the direction and magnitude of diversity effects on microbial life‐history strategies may differ substantially. Future cross‐biome experiments are needed to test the generality of the reported mechanisms.

## 5 Conclusion

Our study provides a robust, mechanistically grounded argument for conserving and restoring plant diversity. By demonstrating that biodiversity not only stabilizes ecosystem processes but can actively enhance microbial efficiency through community sorting and reduced enzymatic costs, we offer a more nuanced perspective on the future of the terrestrial carbon sink. Future research should validate these mechanisms across different biomes and integrate these diversity‐dependent feedbacks into next‐generation Earth‐system models to refine predictions of our planet's climate trajectory.

## Author Contributions

**Dejun Li** designed the research. **Pengpeng Duan**, **Changqing Ye**, **Xinyi Yang**, and **Hu Du** collected data, and performed the fieldwork and lab analysis. Pengpeng Duan conducted all data analyses. Pengpeng Duan wrote the initial draft with significant contribution from Dejun Li, **Chaoqun Wang**, **Hongzhao Yuan**, **Wolfgang Wanek**, **Jian‐Sheng Ye**, and **Kelin Wang**. All authors contributed to the revision of the manuscript.

## Funding

This work was funded by the National Natural Science foundation of China (U24A20576, U22A202679, 42471076), the Guangxi Natural Science Foundation (2023GXNSFDA026039 and 2025GXNSFAA069800), and the Guangxi Bagui Young Scholars Special Funding given to Pengpeng Duan.

## Ethics Approval and Consent to Participate

Not applicable.

## Consent for Publication

Not applicable.

## Conflicts of Interest

The authors declare no conflicts of interest.

## Supporting information




**Supporting File 1**: advs76347‐sup‐0001‐SuppMat.docx.

**Supporting File 2**: advs76347‐sup‐0002‐Data.zip.

## Data Availability

The data that support the findings of this study are openly available in Figshare at https://doi.org/10.6084/m9.figshare.28959509.
