# Supplementary material for: High Tree Species Diversity Promotes Thermal Enhancement Response of Microbial Carbon Use Efficiency
Source: Adv Sci (Weinh). 2026 Jul 3:e76347. Online ahead of print. doi: 10.1002/advs.76347 (PMC13334601; doi:10.1002/advs.76347)
Supplement: Supplementary file 1 — Supporting File 1: advs76347‐sup‐0001‐SuppMat.docx. [file ADVS-9999-e76347-s001.docx]

***Supplementary Information for***

**High tree species diversity promotes thermal enhancement response of microbial carbon use efficiency**

**Running Head: Tree diversity modulates microbial thermal adaptation**

**Pengpeng Duan^1,2,#^, Changqing Ye^1,3,#^, Xinyi Yang^1,2,*^, Chaoqun Wang^4^, Wolfgang Wanek^5^, Jian-sheng Ye^6^, Hongzhao Yuan^2^, Hu Du^1,2^, Kongcao Xiao^1,2^, Xunyang He^1,2^, Kelin Wang^1,2^, Dejun Li^1,2,**^**

^1^Guangxi Key Laboratory of Karst Ecological Processes and Services, Huanjiang Observation and Research Station for Karst Ecosystems, Chinese Academy of Sciences, Huanjiang, Guangxi, China

^2^Institute of Subtropical Agriculture, Chinese Academy of Sciences, Changsha 410125, China

^3^School of Resources and Environmental Engineering, Anhui University, Hefei 230601, China

^4^Ministry of Agriculture and Rural Affairs Key Laboratory of Arable Land Conservation (North China), College of Land Science and Technology, China Agricultural University, Beijing 100193, China

^5^Division of Terrestrial Ecosystem Research, Department of Microbiology and Ecosystem Science, Center of Microbiology and Environmental Systems Science, University of Vienna, Vienna 1010, Austria

^6^State Key Laboratory of Grassland Agro-Ecosystem, College of Ecology, Lanzhou University, Lanzhou 730000, China

**#These authors contributed equally: Pengpeng Duan, Changqing Ye**

***Correspondence (X. Yang & D. Li), Email: Yangxy0804@isa.ac.cn; lidejun@hotmail.com**

**This supplementary file includes:**

Supplementary Methods S1 to S3

Figures S1 to S11

SI References

**Supporting Information Text**

**Supplementary Methods S1: Nuclear magnetic resonance spectroscopy**

The air-dried soils were pretreated with 10% hydrochloric acid (HCl) to remove calcium carbonate and 10% hydrofluoric acid (HF) to eliminate minerals. Following this pretreatment, the soil samples were analyzed using solid-state magic-angle spinning NMR (Bruker DSX 200, Karlsruhe, Germany). To identify specific organic matter functional groups, the ^13^C cross polarization with total suppression of sidebands by adiabatic separation (TOSS) technique was employed. The experiment utilized a 4 mm double-resonance magic angle spinning (MAS) probe operating at a frequency of 100 MHz for ^13^C-NMR. The ^13^C-CP/TOSS experiment was conducted with a spinning speed of 5 kHz, a delay time of 0.8 seconds, a 90° ^1^H pulse width of 4 µs, and a cross-polarization contact time of 1 ms. To enhance resolution, four-pulse total suppression of sidebands and double-pulse phase-modulation decoupling were applied prior to detection. The relative abundance of each functional group was determined by integrating all NMR spectra obtained in current study. The different carbon components were divided into seven chemical shift regions: alkyl C (0–45 ppm), O-alkyl C (45–110 ppm), aromatic C (110–165 ppm), and carbonyl C (165–210 ppm).

**Supplementary Methods S2: SOM fractionation**

All soils were separated after aggregate dispersion into two size fractions: particulate organic matter (POM, >53 μm) and mineral–associated organic matter (MAOM, <53 μm) by wet sieving (1). Sodium hexametaphosphate solution (80 mL) was added to 10 g of air dried, 2 mm sieved soil and shaken for 18 h to disperse soil aggregates. MilliQ water was used to wash the contents through a 53 μm sieve. The two separated fractions were then dried to constant mass at 60 °C. All POM and MAOM fractions were ground and homogenized. Then subsamples were analyzed for C content by dichromate redox colorimetric method. SOC physical stability was expressed as the ratio of MAOC to POC.

To assess the C fraction protected by different minerals, we measured the concentrations of OC associated with calcium (Ca–OC) and iron oxides (Fe–OC). Wang*, et al.* (2) proposed that Ca tightly bound in the Fe-OC-Ca ternary co-precipitates cannot be extracted by mild Na_2_SO_4_ extraction but will be released together with Na_2_SO_4_-extractable Ca (Ca_s_) during strong Fe reduction under CBD treatment. That is, CBD-extractable Ca (Ca_d_) should include Ca_s_ and exceed Ca_s_ in the presence of Ca tightly bound in the Fe-OC-Ca co-precipitates. Similar contents of Ca_s_ and Ca_d_, and OC_CBD_ is positively related to OC_Na2SO4_, indicating efficient extraction of Ca_s_ by the CBD method. Therefore, OC_CBD_ includes OC_Na2SO4_ (Ca–OC), and OC_non–Ca_ (Fe–OC) is calculated as:

$$OC_{non-Ca}=OC_{CBD}-OC_{Na2SO4}\text{ (1)}$$

**Supplementary Methods S3: Soil DNA extraction, high-throughput DNA sequencing and bioinformatics analysis**

Microbial DNA was extracted from 0.5 g aliquots of soil samples at the end of the 365-day incubation using the DNeasy PowerSoil Pro kit (MoBio Laboratories, Carlsbad, CA, USA) according to the manufacturer’s instructions. DNA quality and concentration were assessed through 1.0% agarose gel electrophoresis and measured with a NanoDrop® ND–2000 spectrophotometer (Thermo Scientific Inc., USA). For bacteria, the V3–V4 region of 16S rRNA gene were amplified with primers 338F (5’-ACTCCTACGGGAGGCAGCAG-3’) and 806R (5’-GGACTACHVGGGTWTCTAAT-3’), while fungi were targeted using primers ITS1 (5ʹ-CTTGGTCATTTAGAGGAAGTAA-3ʹ) and ITS2 (5ʹ-TGCGTTCTTCATCGATGC-3ʹ). Sequencing was carried out on an Illumina Miseq 250 platform (Illumina Inc., California, USA). The raw sequence data obtained from amplicon sequencing underwent quality filtering, merging, and clustering to generate Operational Taxonomic Units (OTUs) for both bacterial and fungal communities, with a 97% similarity threshold using the QIIME2 pipeline.


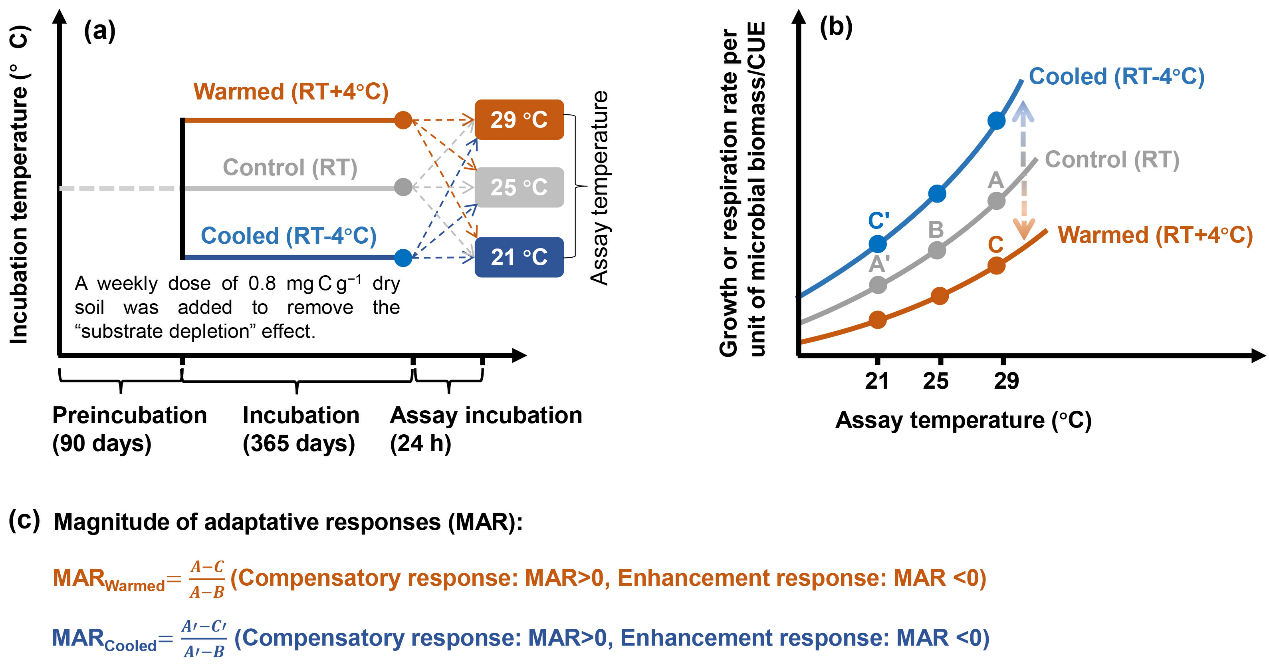


**Figure S1.** Temperature–dependent soil microbial metabolic parameters under warming and cooling conditions. (a) Experimental timeline is divided into three phases: preincubation, incubation, and assay incubation. A weekly dose of 0.8 mg C g⁻¹ dry soil was added to mitigate the “substrate depletion” effect. The 90-day aerobic preincubation period allowed for elimination of sampling disturbance and restore prestress conditions, as well as the stabilization of microbial communities and functions. On day 90, three different thermal treatments, i.e., warmed, cooled and control conditions were established. At the end of the incubation period (day 365), short-term assays (24 hours) were conducted at all three temperatures for each treatment to assess the thermal response of microbial metabolic parameters under sufficient C substrate. (b) Quantifying the thermal response of microbial metabolism parameters to warming and cooling. Growth and respiration rates are expressed per unit of microbial biomass. The reference temperature (RT) was set at 25 °C to represent the mean growing season temperature in the selected forests. The thermal response curve for control soil was generated using microbial metabolic parameters (A', B, and A) assayed at 21, 25, and 29 °C. Similarly, the thermal response curves for warmed and cooled soils were based on parameters (C and C') assayed at 29 °C and 21 °C, respectively. (c) Magnitude of adaptive response (MAR) approach was applied to derive two thermal response metrics, which quantify the thermal response of microbial metabolic parameters to warmed or cooled conditions. An MAR ˃ 0 indicates a compensatory response (reduction of process), while an MAR ˂ 0 suggests an enhancement response (stimulation of process). Higher MAR values indicate stronger compensatory response, whereas lower MAR values indicate stronger enhancement response.


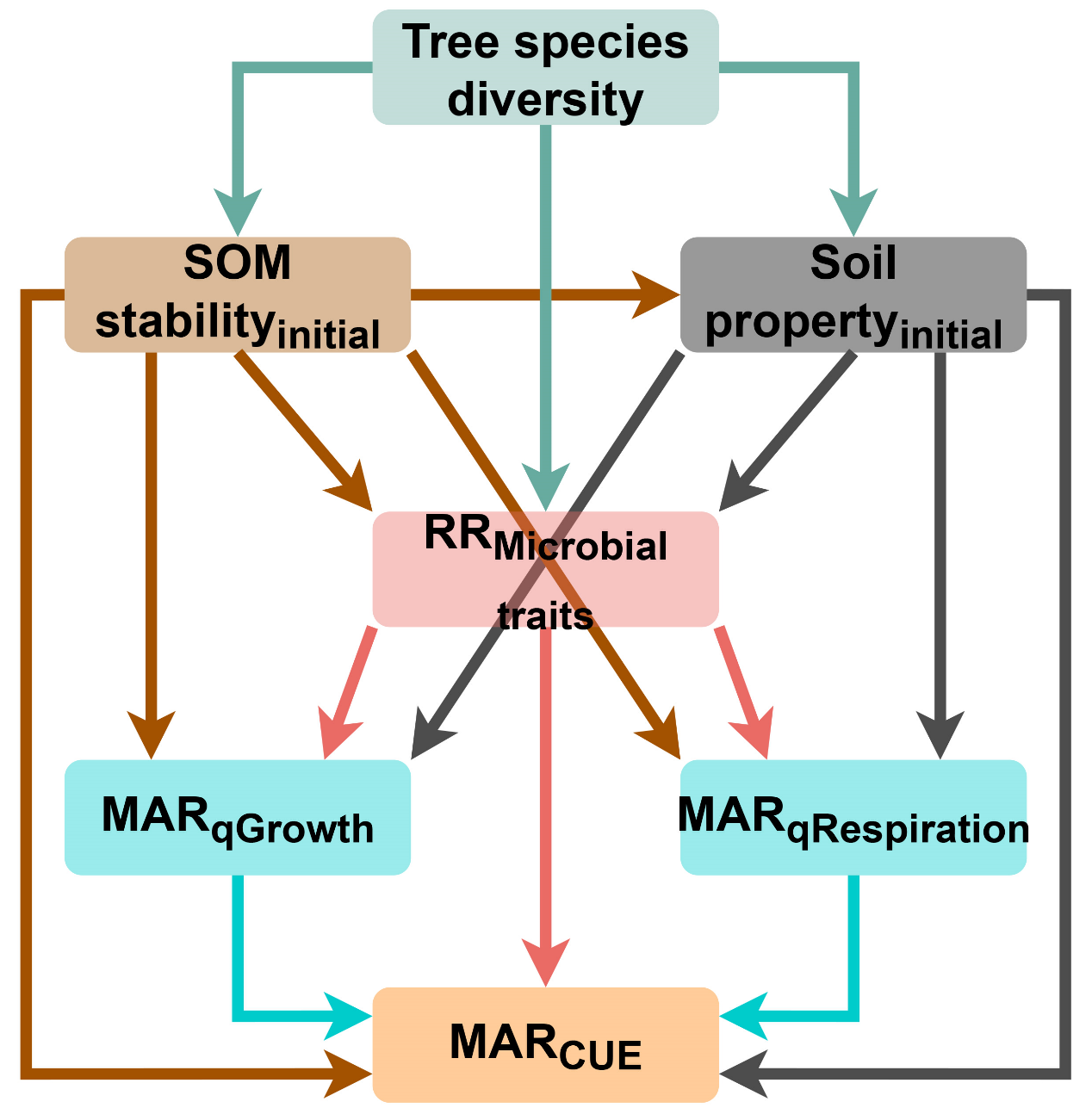
**Figure S2.** *A priori* structural equation model illustrating the direct and indirect effects of tree species diversity on the magnitude of adaptive responses (MAR) of microbial carbon metabolic parameters. The category soil organic matter (SOM) stability defined by chemical recalcitrance, and clay/mineral protection. Soil properties included pH, soil organic carbon, total nitrogen and phosphorus content and their stoichiometry. Microbial traits represented by response ratios (RR) of extracellular enzyme activities, microbial community α-diversity, network complexity, life-history strategies, and negative and positive cohesions. CUE, carbon use efficiency.


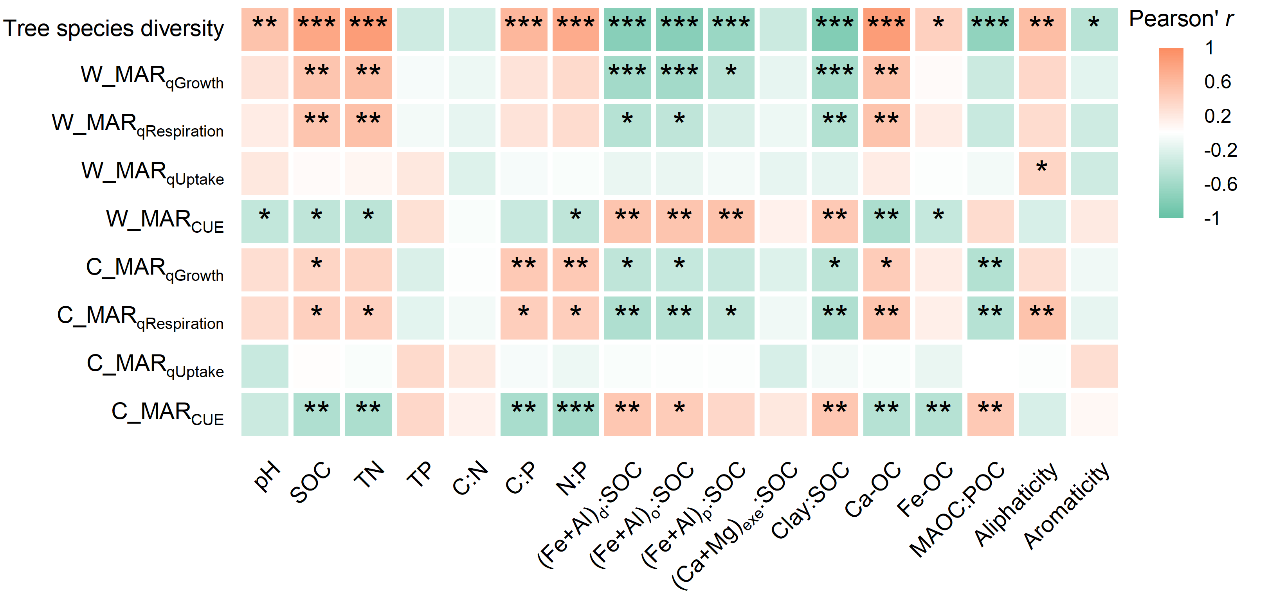


**Figure S3.** Heat map showing the correlations of the soil geochemical properties, and soil organic matter stability indices with tree species diversity, and magnitude of adaptive responses (MAR) of microbial metabolic parameters. Color represents the strength and direction of the Pearson correlation, with the significance levels denoted as follows: **p* < 0.05; ** *p* < 0.01; and *** *p* < 0.001. **Note:** (Fe+Al)_d_:SOC, (Fe+Al)_o_:SOC, (Fe+Al)_p_:SOC, (Ca+Mg)_exe_:SOC, MAOC:POC, clay:SOC, Ca-SOC, Fe-SOC, MAOC:POC, and aromaticity are indicators of higher SOM stability (reflecting greater mineral protection or chemical recalcitrance); aliphaticity is an indicator of lower chemical recalcitrance.


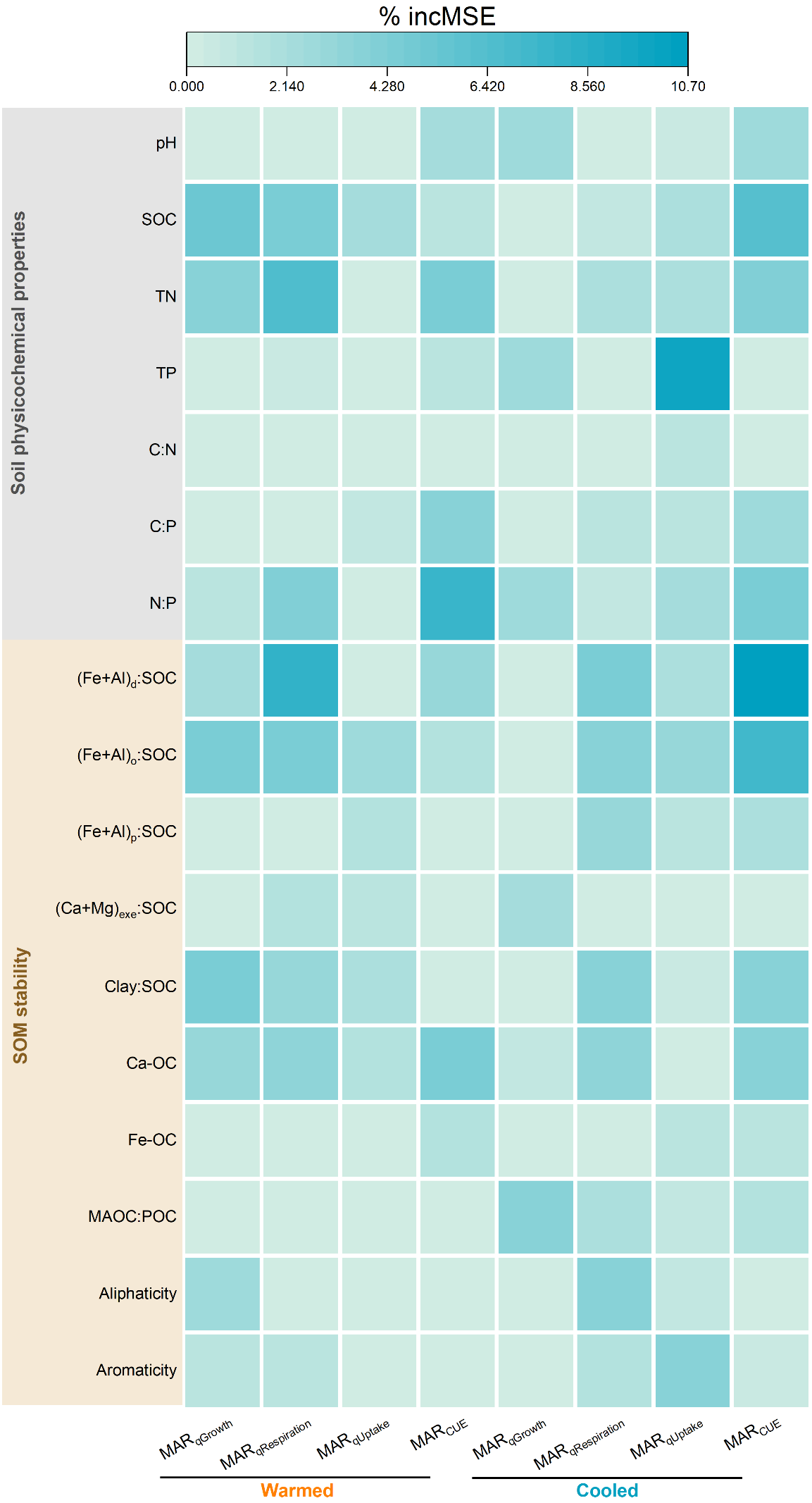


**Figure S4.** Heat map from Random Forest models showing the mean predictor importance (% increase in mean square error) of soil geochemical and organic matter stability indices in predicting the magnitude of adaptive responses (MAR) of microbial metabolic parameters. Color represents the strength of the importance.


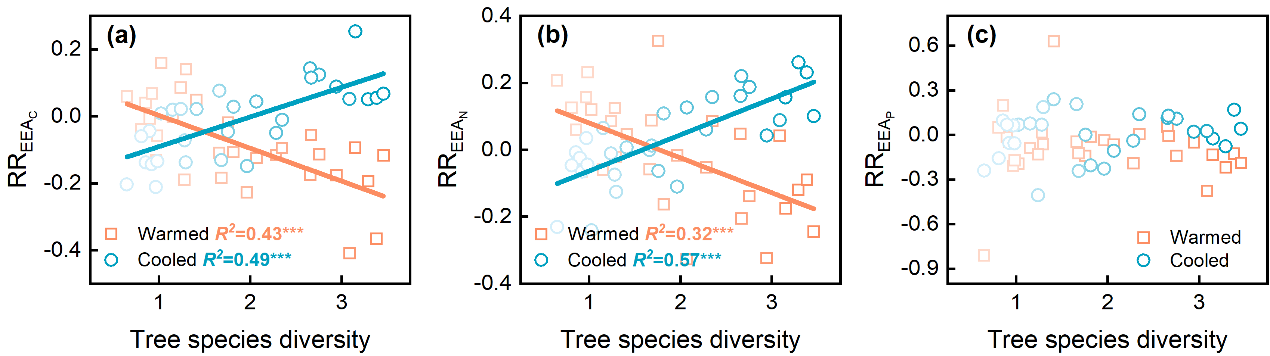


**Figure S5.** Relationships between response ratios (RR) of enzyme activities and tree species diversity. EEA_C_, EEA_N_, and EEA_P_, C, N, and P–acquiring enzyme activities, respectively. Significance is indicated by **p* < 0.05; ** *p* < 0.01; and *** *p* < 0.001.


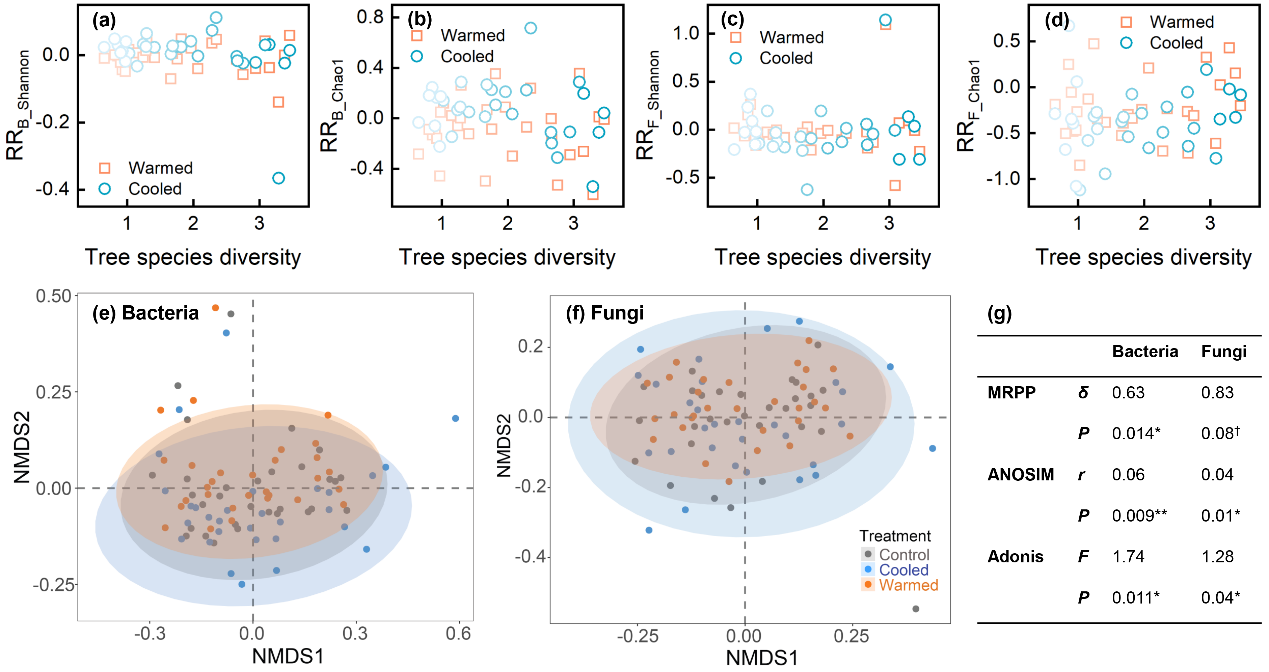


**Figure S6.** Relationships between the response ratios (RR) of microbial α-diversity and tree species diversity (a-d), and the effects of incubation scenarios on the microbial β-diversity (e-g). The incubation scenarios are reference temperature (RT, control), cooled (RT-4 °C), and warmed (RT+4 °C). e-f, Nonmetric multidimensional scaling (NMDS) plots showing the microbial community structure for soil bacteria and fungi. The 95% confidence ellipses are shown around the samples grouped based on different incubation scenarios. g, Significance tests of the networked communities under different incubation scenarios. Three different permutation tests were performed by the multiple-response permutation procedure (MRPP), analysis of similarity (ANOSIM) and Adonis on the basis of Bray–Curtis distance. Significance is indicated by ^†^*p* < 0.1; **p* < 0.05; ** *p* < 0.01; and *** *p* < 0.001.


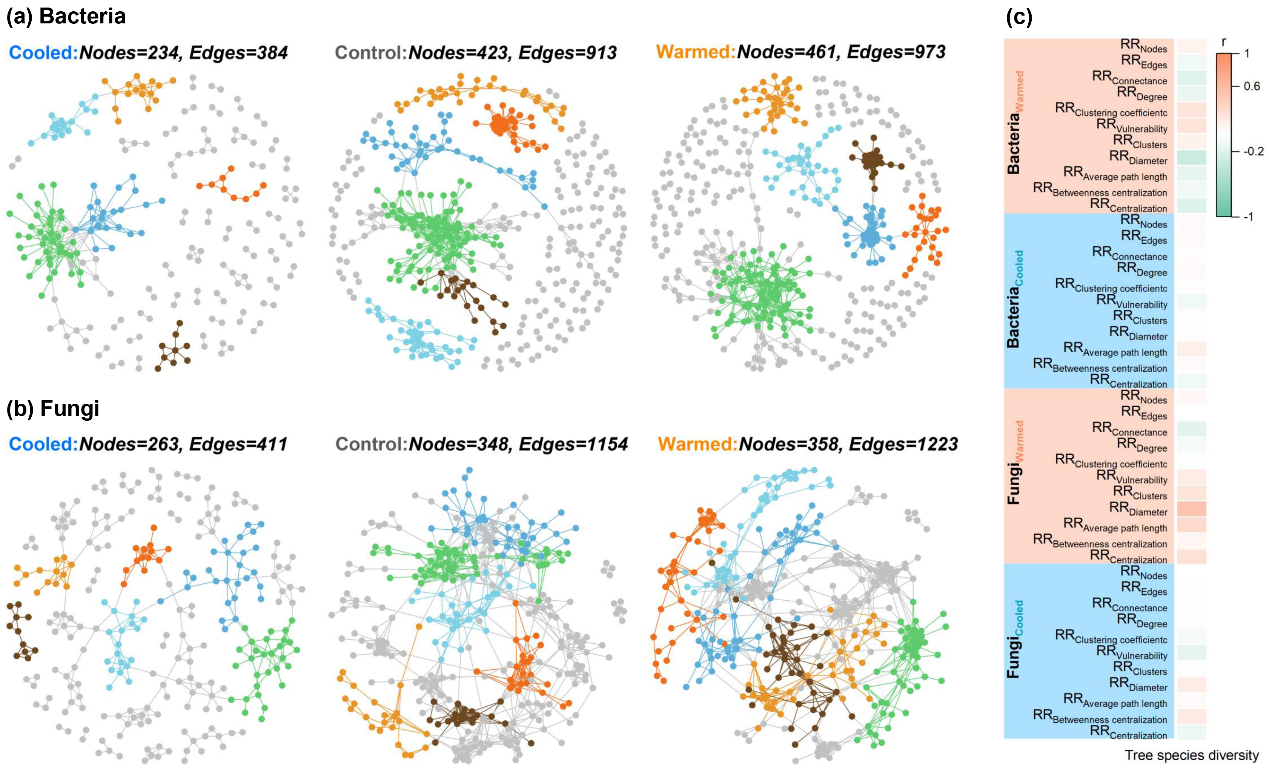


**Figure S7.** Soil microbial networks under different incubation scenarios. a-b, Visualization of constructed molecular ecological networks. Large modules with ≥5 nodes are shown in different colors, and smaller modules are shown in grey. c, Heat map showing the correlations of the network complexity indices with tree species diversity. Color represents the strength and direction of the Pearson correlation, with the significance levels denoted as follows: **p* < 0.05; ** *p* < 0.01; and *** *p* < 0.001.


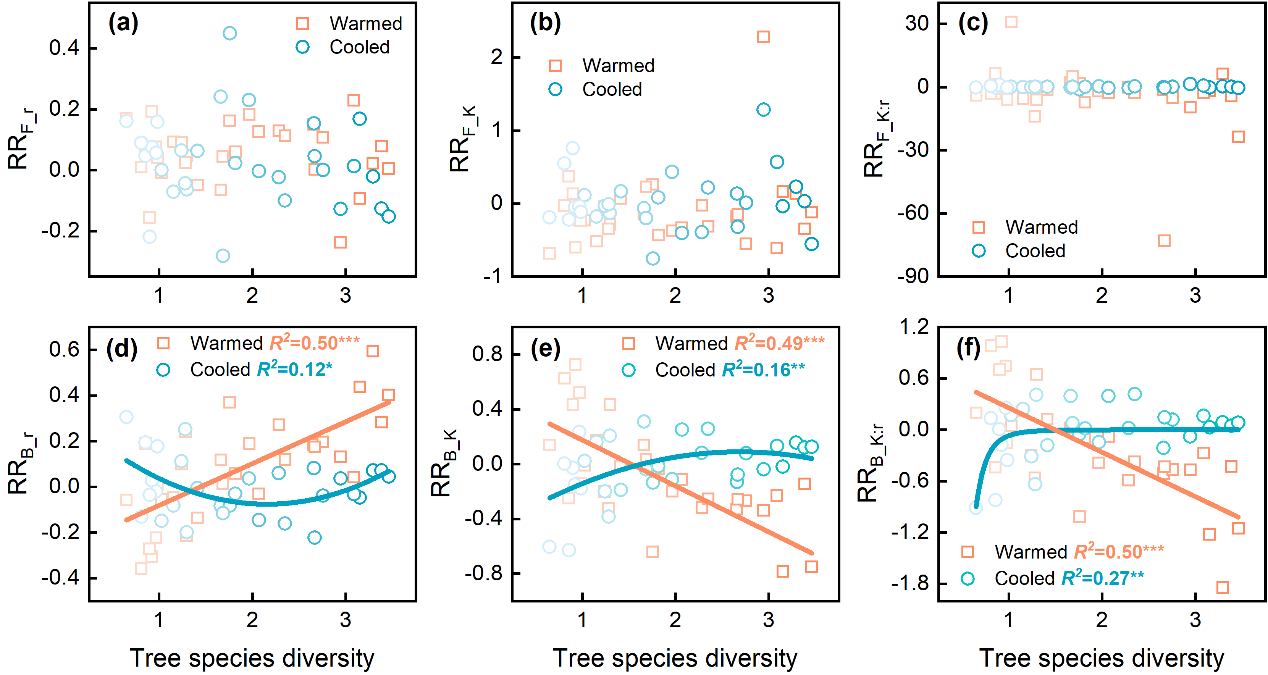


**Figure S8.** The effects of tree species diversity on the response ratios (RR) of soil microbial community composition. Significance is indicated by **p* < 0.05; ** *p* < 0.01; and *** *p* < 0.001.


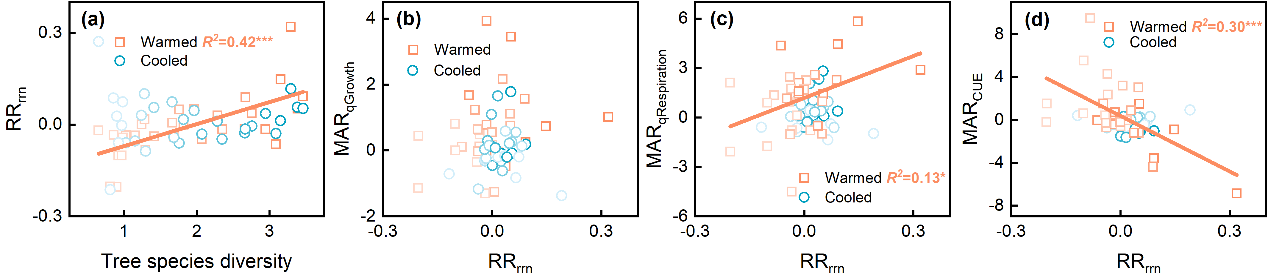


**Figure S9.** Associations among tree species diversity, microbial metabolic adaptation and changes in community level *rrn* copy number of microbial community. Effect of tree species diversity on the response ratios (RR) of *rrn* copy number (a). Relationships between the response ratios (RR) of *rrn* copy number and the magnitude of adaptive responses (MAR) of mass-specific growth (MAR_qGrowth_; b) or mass-specific respiration (MAR_qRespiration_; c) or carbon use efficiency (MAR_CUE_; d). Significance is indicated by **p* < 0.05; ** *p* < 0.01; and *** *p* < 0.001.


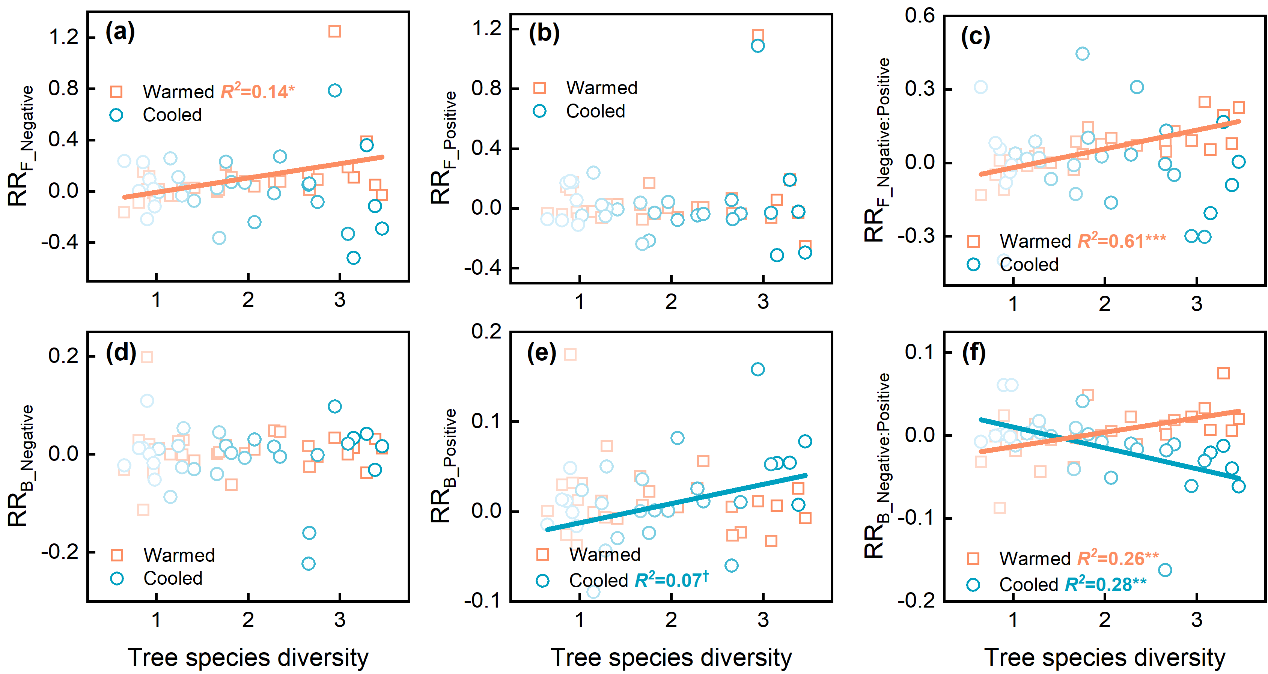


**Figure S10.** The effects of tree species diversity on the response ratios (RR) of soil microbial communities cohesion. Significance is indicated by **p* < 0.05; ** *p* < 0.01; and *** *p* < 0.001.


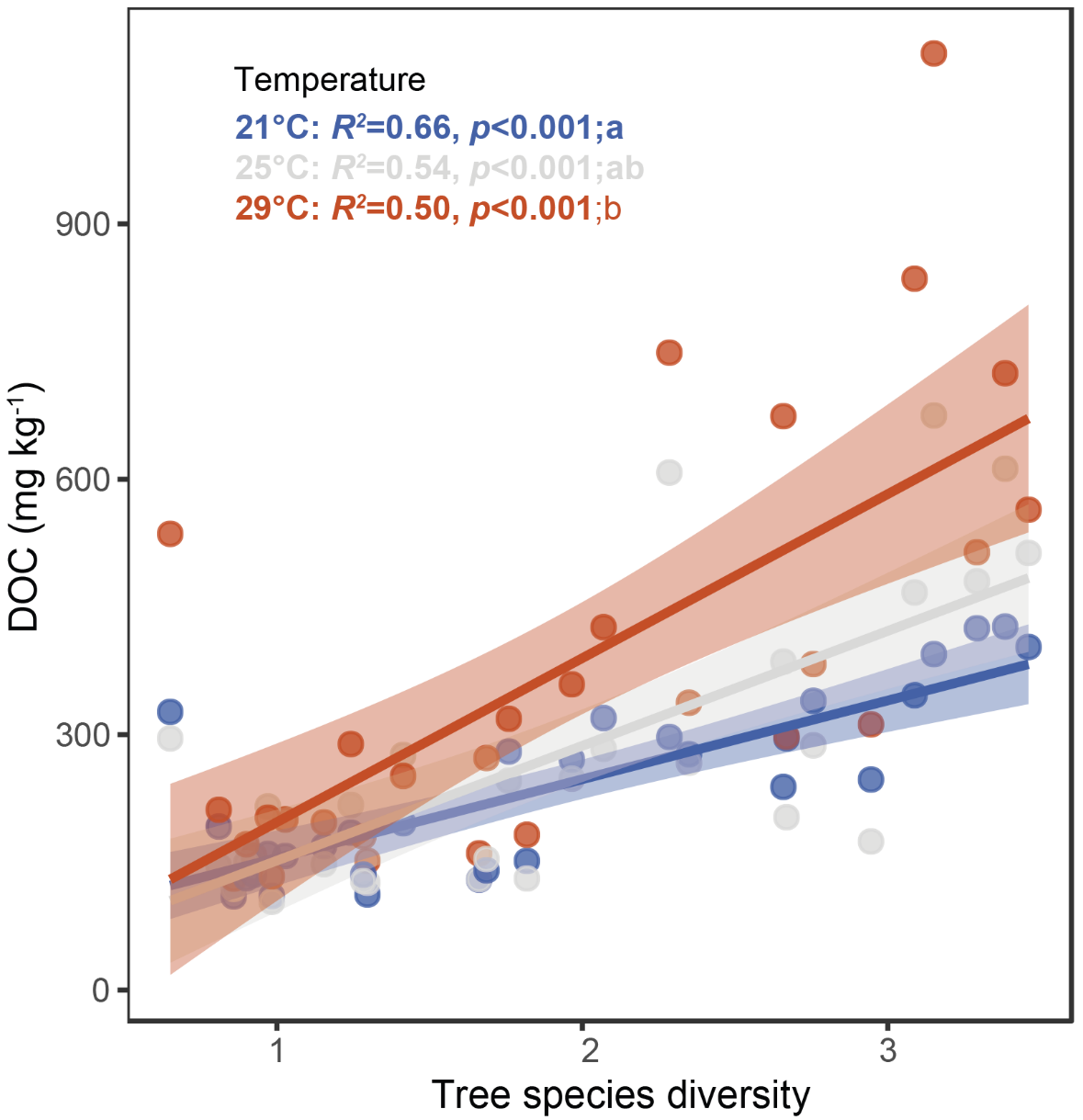


**Figure S11.** **Tree species diversity consistently promotes soil dissolved organic carbon accumulation across an experimental temperature gradient.** Linear regression analysis showing the relationship between tree species diversity and soil dissolved organic carbon (DOC) concentration after a 365 day laboratory incubation under three distinct temperature regimes: 21°C, 25°C, and 29°C. Solid lines represent the fitted ordinary least squares linear regressions, and the semi-transparent shaded bands denote the corresponding 95% confidence intervals. Different lowercase letters adjacent to the regression statistics indicate statistically significant differences between the slopes of the linear regressions at *p*<0.05 based on slope covariance analysis.

**References**

1. Leuthold SJ, Haddix ML, Lavallee J, & Cotrufo MF (2022) Physical fractionation techniques. *Reference Module in Earth Systems and Environmental Sciences*, (Elsevier).

2. Wang S*, et al.* (2021) Delineating the Role of Calcium in the Large-Scale Distribution of Metal-Bound Organic Carbon in Soils. *Geophysical Research Letters* 48(10):e2021GL092391.
